# Hold me or stroke me? Individual differences in static and dynamic affective touch

**DOI:** 10.1371/journal.pone.0281253

**Published:** 2023-05-23

**Authors:** S. Hasan Ali, Adarsh D. Makdani, Maria I. Cordero, Aspasia E. Paltoglou, Andrew G. Marshall, Martyn J. McFarquhar, Francis P. McGlone, Susannah C. Walker, Paula D. Trotter

**Affiliations:** 1 Institute of Population Health, Department of Psychological Sciences, University of Liverpool, Liverpool, United Kingdom; 2 Research Centre for Brain & Behaviour, School of Psychology, Liverpool John Moores University, Liverpool, United Kingdom; 3 Department of Psychology, Manchester Metropolitan University, Manchester, United Kingdom; 4 Institute of Life Course and Medicine Sciences, Pain Research Institute, University of Liverpool, Liverpool, United Kingdom; 5 Walton Centre NHS Foundation Trust, Liverpool, United Kingdom; 6 Neuroscience and Experimental Psychology, The University of Manchester, Manchester, United Kingdom; Universita degli Studi di Pisa, ITALY

## Abstract

Low-threshold mechanosensory C-fibres, C-tactile afferents (CTs), respond optimally to sensations associated with a human caress. Additionally, CT-stimulation activates brain regions associated with processing affective states. This evidence has led to the social touch hypothesis, that CTs have a key role in encoding the affective properties of social touch. Thus, to date, the affective touch literature has focussed on gentle stroking touch. However, social touch interactions involve many touch types, including static, higher force touch such as hugging and holding. This study aimed to broaden our understanding of the social touch hypothesis by investigating relative preference for static vs dynamic touch and the influence of force on these preferences. Additionally, as recent literature has highlighted individual differences in CT-touch sensitivity, this study investigated the influence of affective touch experiences and attitudes, autistic traits, depressive symptomology and perceived stress on CT-touch sensitivity. Directly experienced, robotic touch responses were obtained through a lab-based study and vicarious touch responses through an online study where participants rated affective touch videos. Individual differences were determined by self-report questionnaire measures. In general, static touch was preferred over CT-non-optimal stroking touch, however, consistent with previous reports, CT-optimal stroking (velocity 1–10 cm/s) was rated most pleasant. However, static and CT-optimal vicarious touch were rated comparably for dorsal hand touch. For all velocities, 0.4N was preferred over 0.05N and 1.5N robotic touch. Participant dynamic touch quadratic terms were calculated for robotic and vicarious touch as a proxy CT-sensitivity measure. Attitudes to intimate touch significantly predict robotic and vicarious quadratic terms, as well as vicarious static dorsal hand touch ratings. Perceived stress negatively predicted robotic static touch ratings. This study has identified individual difference predictors of CT-touch sensitivity. Additionally, it has highlighted the context dependence of affective touch responses and the need to consider static, as well as dynamic affective touch.

## Introduction

It has been more than three decades since the discovery of low-threshold mechanosensory C-fibre afferents in the hairy skin of humans, known as C-tactile afferents (CTs), the preferred stimulus for which is a low force, low velocity stroking touch, delivered at skin temperature [1, 2]. Discriminative aspects of touch are encoded by fast conducting Aβ afferents, associated with somatosensory cortex activation, whereas slowly conducting CTs activate the dorsal posterior insular cortex [3, 4], with subsequent processing activating the medial prefrontal cortex, dorsal anterior cingulate cortex, and the mid-anterior orbitofrontal cortex [5, 6]. These brain regions are associated with processing affective states, particularly reward, suggesting CTs are part of a distinct and dedicated pathway for the processing of the positive affective value of touch [1, 7–9].

This assertion is supported by identification of a strong positive correlation between ratings of perceived touch pleasantness and CT mean firing frequency, determined using the single-unit electrophysiological recording technique, microneurography [1]. CTs fire optimally to stroking velocities within the 1–10 cm/s range with average pleasantness ratings plotted against velocity producing an inverted-U function [1]. The tuning of CT afferents to velocity and temperature, combined with the association between CT activation and activation of brain regions associated with affective processing, has led to the proposal of a ‘Social Touch’ hypothesis [10, 11] where CTs play an important role in encoding the rewarding properties of interpersonal social touch.

Social touch involves a variety of gestures delivered at different forces. For example, hugging touch is generally associated with a higher force of touch than caressing touch. Despite the proposed association between social touch and CT activation, relatively little is known about the effect of force on CT responses. Whilst there is some evidence of increased CT firing frequency with increasing force for low force touch of up to 0.1 N [12–14], no such effect has been shown when delivering touch at slightly higher forces of 0.2 N and 0.4 N [1]. Furthermore, there is no evidence regarding how CTs respond to even higher force touches, such as those greater than 0.4 N. However, previous literature has identified CTs respond similarly to both blunt and sharp stimuli [8, 15]. Sharp stimuli will produce a more focal force. This evidence suggests CTs may reach their peak firing potential at relatively low forces, perhaps indicating CTs would respond equally well to light and deep pressure touch.

Social touch gestures include static (e.g. hugging and holding), as well as dynamic, caressing touch. Close static contact is found in several intimate social settings, such as during cuddling, hugging, comforting a person with a hand on the back and in non-intimate social settings such as hand shaking during a friendly introduction. Mammals are also known to implement close static contact to reduce overall surface area for optimal heat retention [16].

Interestingly, evidence suggests CTs respond to both static and dynamic touch [8, 15, 17]. Additionally, CTs have been reported to be intermediate adapting and thus show sustained firing during static touch [15, 17, 18]. These studies provide support for CTs’ role in the encoding of both static and dynamic touch and are particularly relevant when considering certain social touches, such as hugging and hand holding. Despite this evidence, psychophysical and behavioural assessments directly comparing responses to static vs dynamic touch are currently lacking [14].

Skin-to-skin contact (also known as Kangaroo Care) between mothers and infants has been shown to result in improvements in the health of preterm infants, such as increased weight gain and improvements in lung and heart function [19–21]. Kangaroo Care has also been shown to reduce pain responses in preterm infants during Heel Stick tests [22]. Most importantly, Kangaroo Care research indicates that beneficial effects associated with touch can also occur through static, as well as dynamic touch. Additionally, some of these effects are associated with moderate to high pressure, as compared to the light pressure typically associated with CT activation. Interestingly, recent research has proposed expanding the social touch hypothesis to include deep pressure touch, with oscillating deep pressure identified as similarly pleasant and calming as CT-activating stroking touch, but hypothesised to be encoded through a distinct neuronal pathway [23].

In addition to the distinction between discriminative and affective touch, there also remains a further distinction between peripheral and central encoding of gentle touch. Where mechanoreceptive afferents encode the physical properties of touch, the actual subjective percept of gentle touch is processed centrally and would thus be prone to individual variability. Croy and colleagues report individual differences in the inverted-U function between stroking velocity and touch pleasantness, suggesting affective touch sensitivity varies between individuals [24]. One contributing factor may be touch experiences during childhood, with Devine and colleagues reporting early childhood adversity, associated with lower levels of positive childhood touch (TEAQ) [25], led to differences in hedonic ratings of affective touch in later life [26]. Individual differences in touch experiences and attitudes in childhood and adulthood may therefore explain some of the variability in affective touch responses.

Moreover, mood may explain some of the variability in affective touch responses. More specifically, there is evidence that the serotonergic system is involved in the encoding of affective touch [27], that depression is associated with altered social touch responses [28] and that depression levels can decrease following massage therapy for lower back pain [29]. It should also be noted that CT optimal touch has been proposed to be a stress buffer [30, 31] indicating that stress may induce a need state for affective touch, and thus increase its perceived hedonic value. However, as anhedonia (the near complete absence of interest, enjoyment, and motivation) is associated with depression and stress [32–34], touch pleasantness may be reduced by these conditions. Thus, further investigation into how depression and stress influence affective touch responses is required.

In addition to mood conditions, studies have also identified altered affective touch responses in neurodevelopmental conditions such as Autism Spectrum Conditions (ASC), where deep pressure is known to reduce negative mood and behaviour [35]. Individuals with ASC are also shown to display atypical responses during the vicarious perception of affective touch [36]. In contrast to typically developing individuals, those with ASC are shown to have enhanced responses to non-CT optimal touch verses CT-targeted touch in the primary somatosensory cortex, suggesting atypical sensory cortical hyper-reactivity [37]. In addition, autistic traits have been shown to modulate cortical responses to affective, but not discriminative touch [38]. Furthermore, affective touch awareness, which indicates preference for CT-optimal compared to non-optimal dynamic touch, for brush strokes applied to the left dorsal forearm, was identified to negatively correlate with autistic traits, suggesting that autistic traits influence affective touch responses [39]. This is supported by functional neuroimaging investigations, showing autistic traits correlate negatively with cortical responses to CT-optimal touch in the orbitofrontal cortex and superior temporal sulcus [40]. This is vital as the former area is a key area in hedonic processing. In summary, this would indicate that individual differences, particularly in autistic traits, influence affective touch responses.

The main aim of the study was to broaden our understanding of the social touch hypothesis by investigating relative preference for static vs dynamic touch and the influence of force on these preferences. Additionally, we aimed to determine whether self-reported attitudes towards and experiences of affective touch, autistic traits, perceived stress, and depressive symptomology, could account for some of the individual differences in affective touch responses previously reported. Responses to both directly experienced and vicarious touch were obtained.

As static touch is more socially relevant than CT non-optimal stroking touch (stroking touch delivered at a velocity of < 1 cm/s or > 10 cm/s), we hypothesised a preference for static compared to CT non-optimal touch. Static touches, such as hugs and holding touch are typically associated with higher forces than caressing touch, therefore we hypothesised that static touch would be preferred at a higher force (1.5 N) than dynamic touch. In terms of individual differences in affective touch responses, we hypothesised there would be positive relationships between the following variables: attitudes towards and experiences of affective touch, psychophysical and vicarious ratings of touch, and sensitivity to CT targeted touch. Finally, we hypothesised there would be negative relationships between affective touch pleasantness and stress, depressive symptomology, and autistic traits.

## Materials and methods

### Participants

Two-hundred and fifty-three participants (41 male, 211 female, one non-binary) aged between 18–71 (Mean = 23.07, SD = 9.12) took part in the study. Of these 31 (11 males and 20 females) aged 20–71 (Mean = 39.45, SD = 16.43) attended our laboratory at Liverpool John Moores University (LJMU) and completed the laboratory-based, as well as online aspects of this study.

Participants were recruited using posters displayed around the university campuses, emails to research panel lists, and social media advertisements. Participants for the lab-based study were compensated for their time with a £10 gift voucher. Participants for the online study were given the option to enter a prize draw for a £50 gift voucher on completion of the study. Additionally, undergraduate psychology students were awarded course credits for participation.

The inclusion criteria for the lab-based study were that participants had to be aged 18 or over, with no neurological condition, no skin condition affecting the arms and not taking neurologically active medication. For the online study, the inclusion criteria were that participants were aged 18 or over with normal or corrected to normal vision.

This study received ethical approval from the Liverpool John Moores University Research Ethics Committee (ethical approval: 19/NSP/037), as well as from the Manchester Metropolitan University Health, Psychology and Social Care Research Ethics and Governance Committee (EthOS ID: 10311). Written informed consent was obtained for the lab-based study. Informed implied consent was obtained for the online study through study completion and response submission.

### Materials

#### Touch Experiences and Attitudes Questionnaire

The Touch Experiences and Attitudes Questionnaire (TEAQ) [25] is a 57-item self-report questionnaire. The TEAQ is designed to measure experiences of and attitudes towards positive touch across relationships and the lifespan using six subscales: Friends and Family Touch (FFT), Current Intimate Touch (CIT), Childhood Touch (ChT), Attitude to Self-Care (AtSC), Attitude to Intimate Touch (AIT) and Attitude to Unfamiliar Touch (AUT). Subscale scores are obtained through calculation of a mean score per subscale for the items belonging to each subscale. Participants rate each statement on a 5-point Likert scale ranging from 1-“Disagree strongly” to 5-“Agree strongly”. High scores indicate more positive attitudes towards and experiences of affective touch. The TEAQ has been shown to have high internal consistency (Cronbach Alpha = .78 - .92) [25].

#### Autism Quotient

The Autism Quotient (AQ) [41] is a 50-item self-report questionnaire which aims to assess the level of autistic traits that an individual has over five separate subscales: Social Skill, Attention Switching, Attention to Detail, Communication and Imagination. For half of the items, a ‘definitely agree’ or ‘slightly agree’ response indicates characteristics similar to those on the autistic spectrum and are scored as 1, whereas ‘definitely disagree’ or ‘slightly disagree’ responses are scored as 0. The other half of the questions are reverse scored. Subscale scores are determined through summing the scores of the items belonging to each subscale. A total score is calculated by summing the scores obtained for all 50 items. The minimum total score on the AQ is 0 and the maximum is 50. If an adult has a score equal to or more than 32, this is highly predictive of ASC [41].

#### Patient Health Questionnaire-9

The Patient Health Questionnaire-9 (PHQ-9) [42] is a 9-item questionnaire designed to measure the nine symptoms of depression, as defined by the Diagnostic and Statistical Manual of Mental Disorders, fourth edition (DSM-IV) (American Psychiatric Association, 1994), that an individual has experienced over the last two weeks. Participants rate how often they have been bothered by the symptoms on a 4-point Likert scale ranging from 0-“not at all” to 3-“nearly every day”. In addition to this, participants also rate how difficult they have found it to overcome the symptoms. The PHQ-9 is scored by summing the scores for the nine symptom items. Participants can obtain a minimum score of 0 and a maximum score of 27. A higher score indicates higher depressive symptomology.

#### Perceived Stress Scale

The Perceived Stress Scale (PSS) [43] is a 10-item self-report questionnaire designed to assess participant’s stress experiences over the last month. Participants rate each statement on a 5-point Likert scale ranging from 0-“never” to 4-“very often”. Four items are reverse scored, then a total score is obtained by summing the scores for all items. Scores can range from a minimum of 0 to a maximum of 40, with higher scores representing greater levels of perceived stress.

#### Rotary Tactile Stimulator (RTS)

Robotic touch was delivered using an RTS (Dancer Design. St Helens, UK) with a flat-bottomed probe covered with a soft polyurethane foam pad, with a stroking surface measuring ~10 x 2 cm. Participants experienced six velocities: 0 cm/s (static), 0.3 cm/s, 1 cm/s, 3 cm/s, 10 cm/s and 30 cm/s at three forces: 0.05 N, 0.4 N, and 1.5 N in a randomised manner for a total of 18 trials per block. Participants experienced all 18 different stimuli once per block, with participants experiencing three blocks in total. The RTS applied the touch to the ventral forearm in a proximal to distal fashion over an aperture of approximately 5 cm. Participants rated the perceived pleasantness and intensity of each stroke using a visual analogue scale (VAS). For ratings of touch pleasantness, participants were asked: “Please rate the pleasantness of the sensation”. A visual analogue scale (VAS) was provided beneath the question with anchor points “-10 Unpleasant” on the far left, “0 Neutral” at the mid-point, indicated by a purple point on the scale and “+10 Pleasant” on the far right. For ratings of touch intensity, the question: “Please rate the intensity of the sensation”, with a VAS scale below the question with anchor points “0 Not detectable” on the far left and “+100 Most intense sensation imaginable” on the far right. The VAS scales were displayed on a 43” TV screen (Hitachi, Tokyo, Japan) with a 1920 x 1080 resolution. The TV screen was placed approximately 1.5 meters away from the participant and 1 meter away from the ground on a portable stand. Participants responded using a response slider consisting of a slider which allowed them to move an onscreen cursor and a blue button on the left to confirm the participant’s choice. Using the response slider, participants made their ratings by moving a cursor displayed on the VAS to the position they felt was most appropriate, then confirming their response using the response button on the left. Participants were asked to return the slider back to the midpoint, indicated by the purple mark on the response slider, after each rating.

#### Touch videos rating task

The touch videos used in this study are from the same collection of videos reported previously [44]. Touch videos consisted of a female applying various touches to a male. The videos lacked any form of social context and only the arm and hand of the female and body area of the male being touched was visible. The male was stroked at velocities of 30 cm/s, 3 cm/s, 0.5 cm/s, as well as 0 cm/s, static touch. All videos were six seconds long and depicted touch being applied to either the upper arm, dorsal forearm, ventral forearm, dorsal hand or palm. All videos were presented in a randomised order with one velocity and location depicted per video, leading to twenty videos in total. Participants were asked two questions after each video: “How pleasant do you think the action was for the person being touched?”, where participants responded on a 7-point Likert scale ranging from “1-very unpleasant” to “7-very pleasant”, followed by “How much would you like to be touched like this?” to which participants responded on a 7-point Likert scale ranging from “1-not at all” to “7-very much so.

### Procedure

For the laboratory-based study, after providing written informed consent, participants completed a screening questionnaire to verify they met the inclusion criteria for the study and provided demographic details, including their age and gender. They then completed a paper-based PHQ-9. Following this, the robotic touch protocol using the RTS was carried out. Participants were sat in a dental chair with their left forearm fixed with a VacFix® (Par Scientific) cushion to prevent movement. Once the RTS was calibrated, participants were given a trial session of the RTS, where the researcher explained how to provide responses to the touch and participants provided some practice ratings. Once the trial session was complete and participants were able to use the response box successfully, the first block was started. Before each stroke, participants were asked to close their eyes and then asked to open their eyes and rate the stroke once it had finished. For static touches, participants were told by the researcher (SHA) when to open their eyes to rate the touch, which was after the touch had been applied for three seconds. A static touch duration of three seconds was chosen to reflect the average duration of naturally occurring static touches, such as embraces, which have been reported to have a mean duration of three seconds [45]. Additionally, we have previously applied three second static touches, due to longer duration static touches feeling unnatural [46]. After the first block, participants were then seated at a desk and completed the touch videos rating task described above. Following completion, participants were taken back to the dental chair where the RTS was re-calibrated, and the second stroking block began. Participants were then seated at the desk again to complete the AQ. They then returned to the dental chair a final time to complete block three of the RTS task. Finally, participants returned back to the desk to complete the TEAQ and PSS. The touch videos, AQ, TEAQ and PSS were all completed via Qualtrics^®^ (Provo, UT, USA). Participants were debriefed at the end of the study.

For the online study, participants followed a link whereby they were presented with the Participant Information Sheet using Qualtrics®. Participants were then screened for eligibility. If eligible to take part, they were then asked to provide consent and provide demographic information, including their age and gender. Participants were then asked to complete the touch videos rating task described above. Following this, participants completed the AQ followed by the PSS, TEAQ and PHQ-9. Participants were then debriefed.

### Data analysis

Examination of histograms and QQ-norm plots of model residuals revealed the data to be normally distributed. As participant ratings were on a continuous scale and our data met the assumptions for parametric analyses [47, 48], ratings for both robotic touch and vicarious touch were analysed using a linear mixed-effects model fit using the lmer function from the lme4 package [49] in R [50].

For robotic touch, the dependent variable was the pleasantness/intensity ratings, with fixed effects of force with three levels: 0.05, 0.4 and 1.5 N, and velocity with 6 levels: 0, 0.3, 1, 3, 10 and 30 cm/s. A random effect of participant was included in the model. One female participant was excluded from this analysis due to an incomplete dataset.

For vicarious touch responses, the dependent variable was the ratings provided, with three fixed effects: question with two levels (self vs other focus), touch velocity with four levels (0, 0.3, 3, and 30 cm/s) and touch location with five levels (upper arm, dorsal forearm, ventral forearm, dorsal hand and palm). A random effect of participant was included in the model.

For both models, omnibus effects were tested using Kenward-Roger F tests using the Anova function from the car package [51]. When required, significant effects were followed up using the *emmeans* function from the emmeans package version 1.6.1 [52] with FDR correction for multiple comparisons.

For the individual differences analyses, the quadratic velocity terms for dynamic touch (excluding static touch) for each participant was calculated for each force separately using R [50]. Log_10_ velocity was used, as the velocities selected for this study were chosen based on their equidistance on a log scale. The above mentioned female participant was excluded from these analyses, as well as one additional female, due to missing data. Additionally, one further female was excluded from analyses using the TEAQ, with two other female participants being excluded from the analyses using the PSS, PHQ-9 and AQ total score, due to missing data.

## Results

### Pleasantness ratings of directly experienced robotic touch

**Fig 1:**
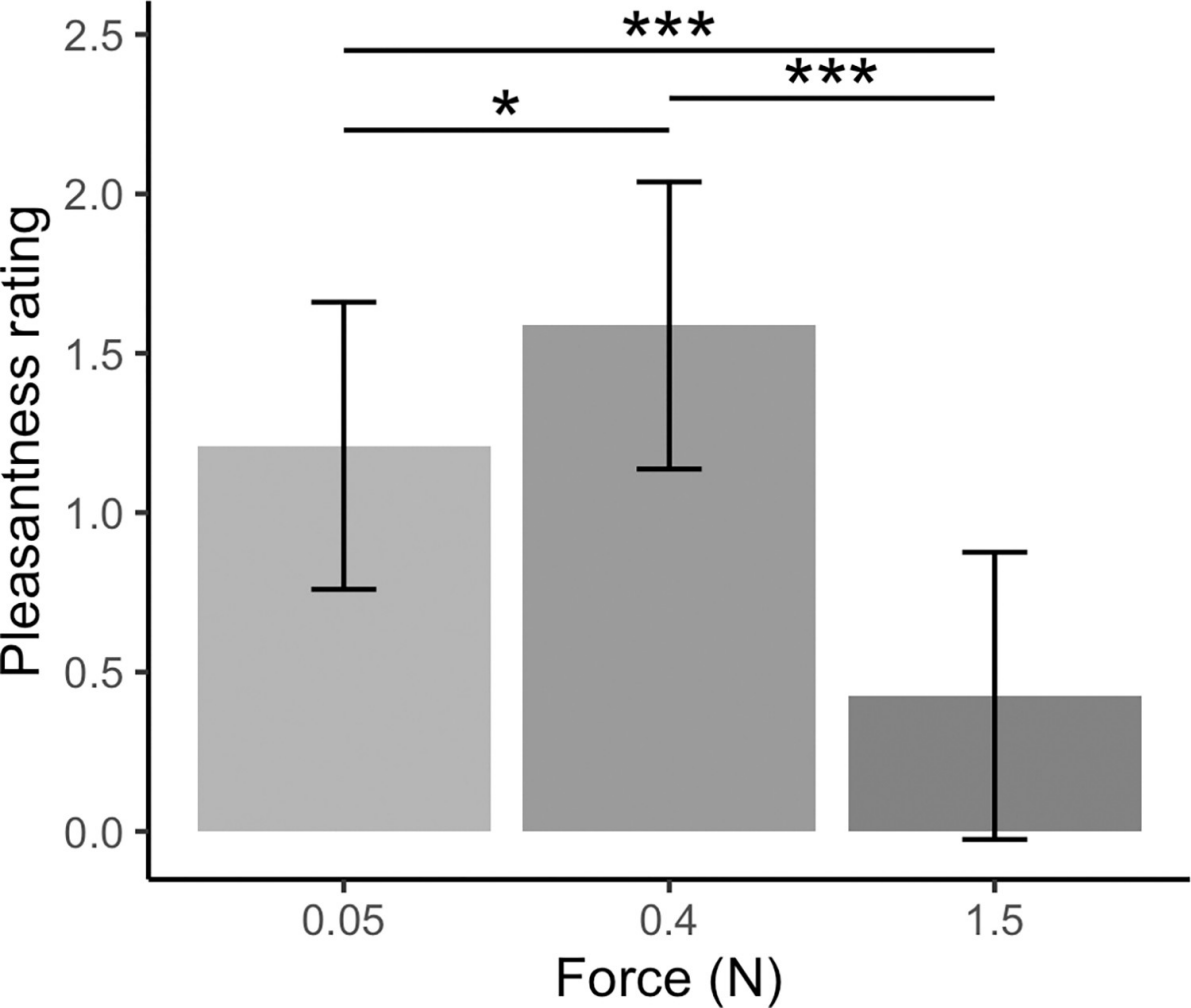
Effect of force on pleasantness ratings for directly experienced, robotic touch to the ventral forearm. Means ± 95% confidence intervals are shown. Touch delivered at a force of 0.4 N was rated significantly more pleasant than touch delivered at 0.05 N (*p* = .021) and 1.5 N (*p* < .001). Touch delivered at 0.05 N was rated significantly more pleasant than touch delivered at 1.5 N (*p* < .001).

The main effect of force was significant (*F*(2, 1609.0) = 26.50, *p* < .001). Pairwise comparisons identified 0.4 N touch, typically used in studies investigating CT-targeted touch, was rated as significantly more pleasant than 0.05 N touch (*t*(1609) = 2.32, *p* = .021) and 1.5 N touch (*t*(1609) = 7.14, *p* < .001). Additionally, 0.05 N touch was rated as significantly more pleasant than 1.5 N touch (*t*(1609) = 4.82, *p* < .001). These findings are summarised in Fig 1.

The main effect of velocity was also significant (*F*(5,1609.7) = 33.08, *p* < .001). Pairwise comparisons revealed 10 cm/s touch to be rated as significantly more pleasant than all other velocities (*t*s ≥ 4.23, *p*s < .001). In partial support of our hypothesis that static touch would be preferred over CT non-optimal dynamic touch, due to the greater social relevance of static touch, significantly greater pleasantness ratings were obtained for static than 0.3 cm/s touch (*t*(1610) = 2.37, *p* = .021) and static touch was rated as significantly less pleasant than the CT optimal velocities of 1, 3 and 10 cm/s (*t*s ≥ 2.37, *p* ≤ .021). However, ratings of static compared to 30 cm/s touch were not significantly different (*t*(1610) = 1.75, *p* = .086).

**Fig 2:**
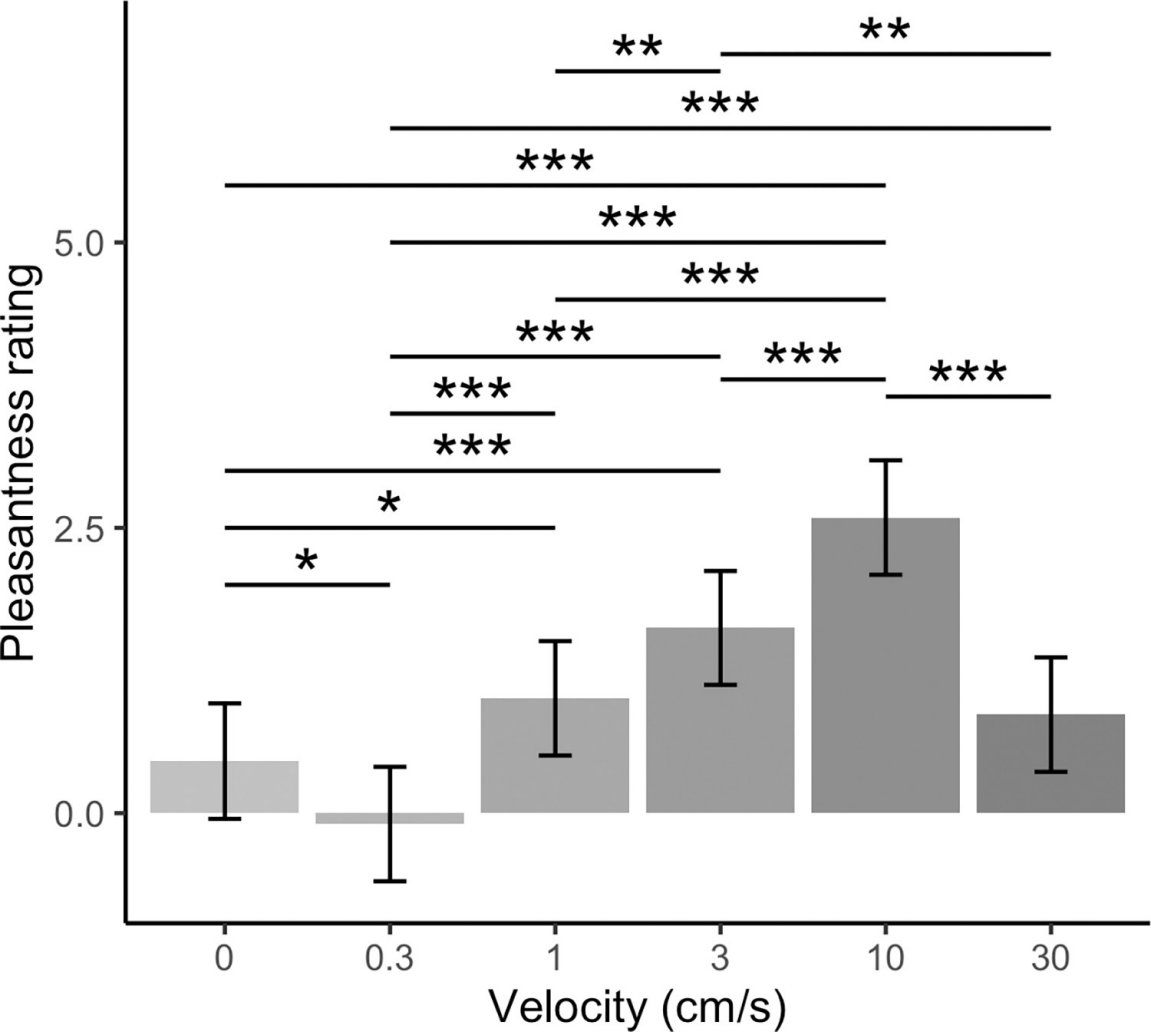
Effect of velocity on pleasantness ratings for directly experienced robotic touch to the ventral forearm. Means ± 95% confidence intervals are shown. Touch delivered at 10 cm/s was rated as significantly more pleasant than all other velocities (*p*s < .001), with 0.3 cm/s touch rated as significantly less pleasant than all other velocities (*p*s ≤ .021). All pairwise comparisons were significant, apart from the difference between static and 30 cm/s touch (*p* = .086) and the difference between 1 and 30 cm/s touch (*p* = .535).

Touch delivered at 0.3 cm/s was rated as significantly less pleasant than touch delivered at 1, 3, 10 and 30 cm/s (*t*s ≥ 4.16, *p*s < .001). Touch delivered at 1 cm/s was rated significantly less pleasant than touch at 3 and 10 cm/s (*t*s ≥ 2.70, *p*s ≤ .010), but there was no significant difference for ratings of touch delivered at 1 compared to 30 cm/s (*t*(1609) = 0.62, *p* = .535). Touch delivered at 3 cm/s was rated as significantly more pleasant than 30 cm/s touch (*t*(1610) = 3.32, *p* = .001). These findings are summarised in Fig 2.

The interaction of force with velocity was not significant (*F*(10,1609.0) = 1.52, *p* = .126). This does not support our hypothesis that static touch may be preferred at a higher force (1.5 N) than dynamic touch. Rather, our results show dynamic and static touch are both preferred at 0.4 N and that a velocity of 10 cm/s is most pleasant.

### Vicarious touch ratings

#### Combined lab and online dataset

The three-way interaction of question by velocity by location was not significant (*F*(12, 9826) = 0.39, *p* = .968). The two-way interactions of question by velocity (*F*(3,9826 = 0.79, *p* = .497) and question by location (*F*(4, 9826 = 0.24, *p* = .913) were also not significant.

The two-way interaction of velocity by location was significant (*F*(12, 9826) = 18.42, *p* < .001). Pairwise comparisons identified that for the palm, 3 cm/s touch was rated significantly more positively than static, 0.5 cm/s and 30 cm/s touch (*t*s(9826) ≥ 4.66, *p*s < .001). Additionally, static touch was rated significantly more positively than 0.5 cm/s touch and 30 cm/s touch (*t*s(9826) ≥ 2.43, *p*s ≤ .015). Touch applied at a velocity of 0.5 cm/s was rated significantly more positively than 30 cm/s touch (*t*(9826) = 7.27, *p* < .001).

For touch applied to the dorsal hand, ratings for touch applied at 3 cm/s and static touch were not significantly different (*t*(9826) = 0.26, *p* = .793). Static and 3 cm/s touch were rated significantly more positively than 0.5 and 30 cm/s touches (*t*s(9826) ≥ 11.12, *p*s < .001). Ratings for 0.5 cm/s touch and 30 cm/s touch were not significantly different (*t*(9826) = 1.12, *p* = .316).

For touch applied to the dorsal forearm, 3 cm/s touch was rated significantly more positively than static, 0.5 cm/s and 30 cm/s touch (*t*s(9826) ≥ 9.15, *p*s < .001). Static touch was rated significantly more positively than 0.5 cm/s and 30 cm/s touch (*t*s(9826) ≥ 5.46, *p*s < .001). Ratings for 0.5 cm/s and 30 cm/s touch were not significantly different (*t*s(9826) 1.67, *p* = .096).

For touch applied to the ventral forearm, 3 cm/s touch was rated significantly more positively than static, 0.5 cm/s and 30 cm/s touch (*t*s(9826) ≥ 9.12, *p*s < .001). Touch applied at 0.5 cm/s was rated significantly more positively than static and 30 cm/s touch (*t*s(9826) ≥ 5.10, *p* < .001). Ratings for static compared to 30 cm/s touch were not significantly different (*t*(9826) = 0.76, *p* = .446).

For touch applied to the upper arm, 3 cm/s touch was rated significantly more positively than static, 0.5 cm/s and 30 cm/s touch (*t*s(9826) ≥ 9.03, *p*s < .001). Static touch was rated significantly more positively than 0.5 cm/s and 30 cm/s touch (*t*s(9826) ≥ 2.19, *p*s ≤ .034). Ratings for 0.5 cm/s and 30 cm/s touch were not significantly different (*t*(9826) = 0.29, *p* = .775).

**Fig 3:**
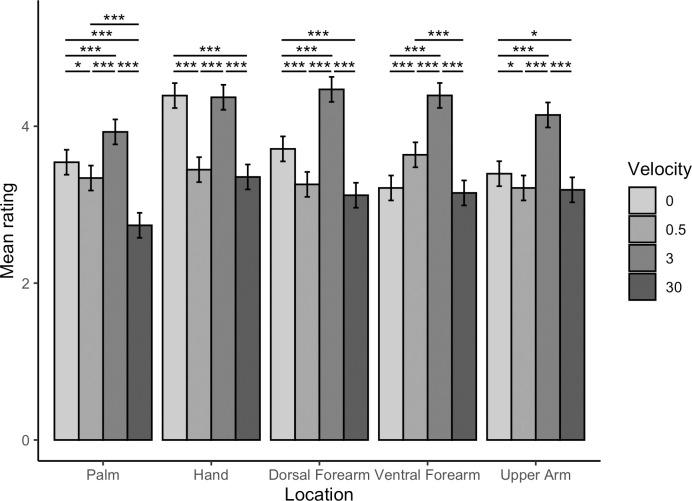
Interaction effect of location by velocity on vicarious touch ratings. Means ± 95% confidence intervals are shown. For touch applied to the dorsal hand, static touch (0 cm/s) was not rated as significantly different to CT-optimal (3 cm/s) touch (*p* = .793). For all other locations, CT-optimal (3 cm/s) touch was rated significantly more pleasant than all other velocities (*p*s < .001).

Thus, at all locations CT optimal touch was rated higher than slower and faster non-CT optimal dynamic touch. However, preference for static touch varied by location, with static touch being rated equally pleasant to CT optimal touch on the dorsal hand. Consistent with our hypothesis that static touch would be preferred over CT non-optimal dynamic touch, static touch was preferred to slow (0.3 cm/s) and fast (30 cm/s) non-CT optimal touch, except on the ventral forearm. These findings are summarised in Fig 3.

**Fig 4:**
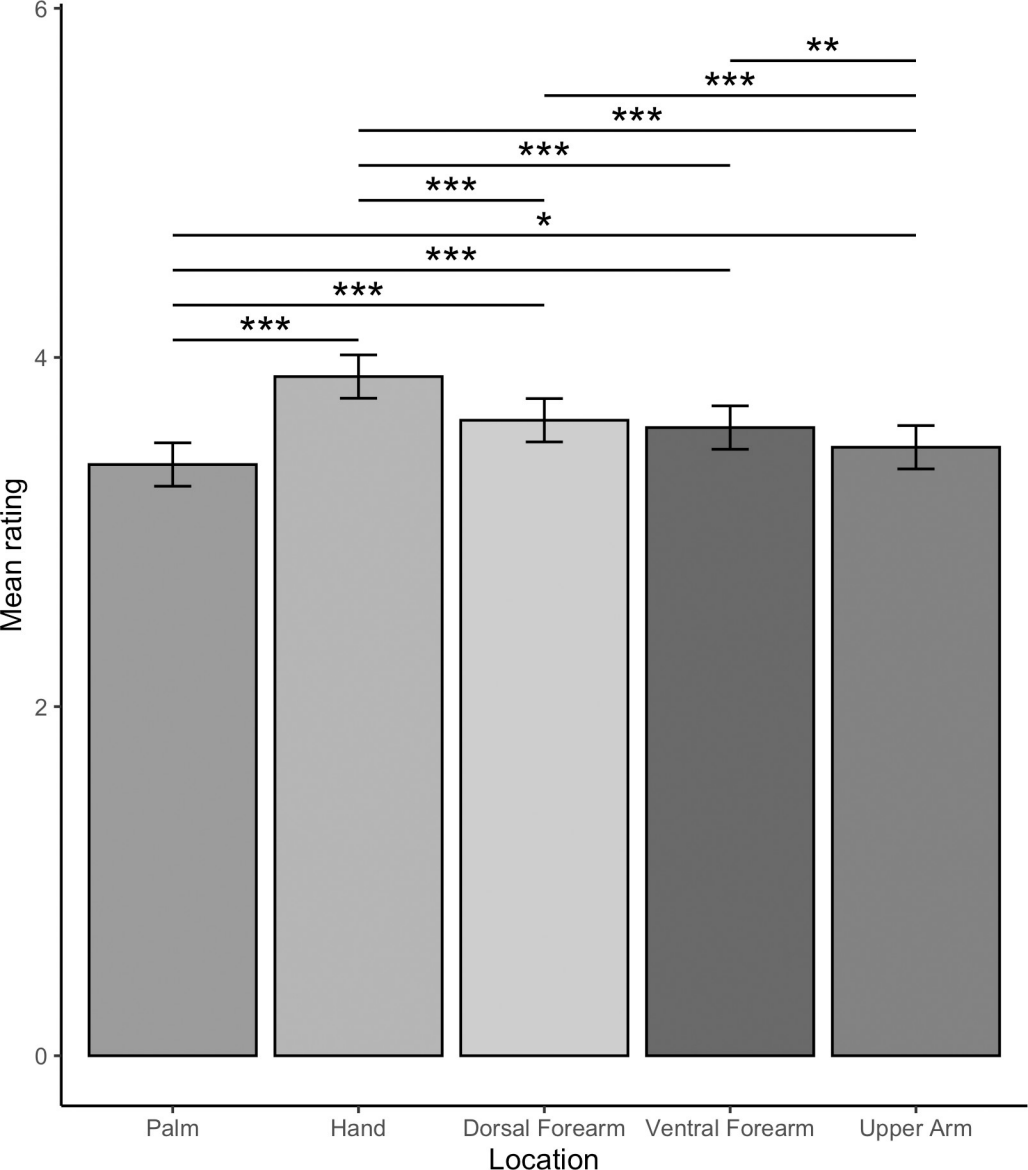
Effect of location on vicarious touch ratings. Means ± 95% confidence intervals are shown. Pairwise comparisons comparing the ratings for each location to all other locations were all significantly different (*p*s ≤ .019), except ratings for the dorsal compared to ventral forearm (*p* = .311).

The main effect of location was significant (*F*(4,9826) = 41.96, *p* < .001). Pairwise comparisons comparing the ratings for each location to all other locations were all significantly different (*t*s(9826) ≥ 2.38, *p*s ≤ .019), except ratings for the dorsal compared to ventral forearm (*t*(9826) = 1.01, *p* = .311). As shown in Fig 4, ratings were highest for touch to the dorsal hand, followed by the forearm then upper arm, with touch to the palm rated least positively.

**Fig 5:**
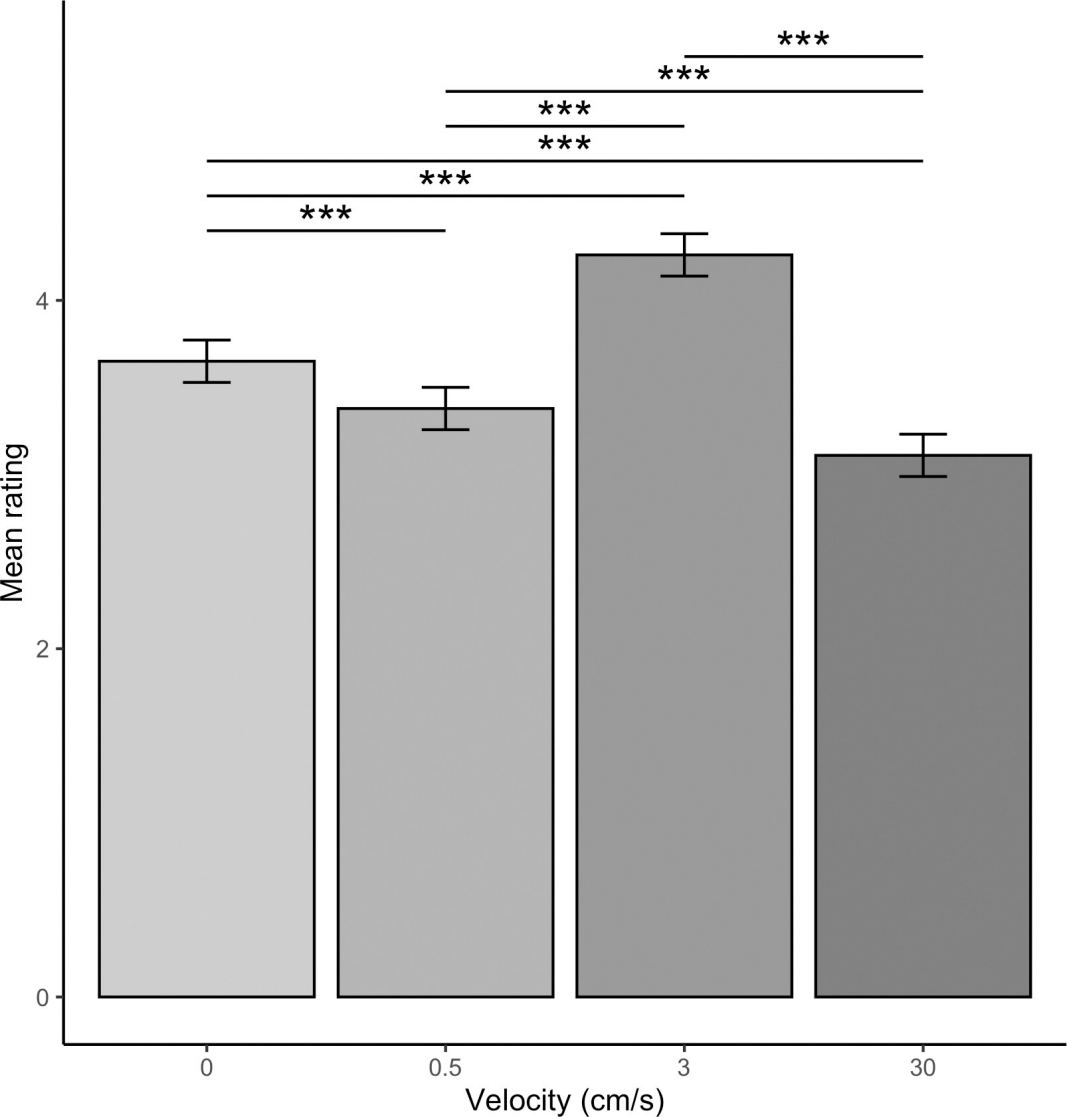
Effect of velocity on vicarious touch ratings. Means ± 95% confidence intervals are shown. Pairwise comparisons identified CT-optimal 3 cm/s touch was rated significantly more positively than CT non-optimal static, 0.5 cm/s and 30 cm/s touch (*t*s(9826) ≥ 16.45, *p*s < .001). However, static touch was rated significantly more positively than 0.5 cm/s touch and 30 cm/s touch (*t*s(9826) ≥ 7.32, *p*s < .001). Touch at 0.5 cm/s was rated significantly more positively than 30 cm/s touch (*t*(9826) = 7.24, *p* < .001).

The main effect of velocity was also significant (*F*(3,9826) = 352.56, *p* < .001). Pairwise comparisons identified CT-optimal 3 cm/s touch was rated significantly more positively than all other velocities (*t*s(9826) ≥ 16.45, *p*s < .001). However, supporting our hypothesis that static touch would be preferred over CT non-optimal dynamic touch, static touch was rated significantly more positively than 0.5 cm/s touch and 30 cm/s touch (*t*s(9826) ≥ 7.32, *p*s < .001). Touch at 0.5 cm/s was rated significantly more positively than 30 cm/s touch (*t*(9826) = 7.24, *p* < .001). These results are summarised in Fig 5.

Additionally, the main effect of question was significant (*F*(1,9826) = 292.20, *p* < .001). Overall, participant ratings for how pleasant the touch was for the person receiving the touch in the video (other-focussed question) were significantly higher than ratings for how much they would like to be touched like that (self-focussed question).

#### Individual differences analyses

*Lab data (robotic touch). Effect of force.* There was no significant effect of force on the participants’ quadratic velocity terms (*F*(2,60) = 1.27, *p* = .288). Examination of the QQ norm plot and histogram of model residuals identified one participant as an outlier. After removal of this participant, the effect of force was still not significant (*F*(2,58) = 2.67, *p* = .078), but the QQ norm plot and histogram identified no further outliers. It was therefore decided to investigate how individual difference measures predicted the quadratic velocity term for each participant for the optimally rated 0.4 N force, commonly used in previous CT research. A mixed-effects model was not required, as there was only one data point per participant, so the *lm* function was used in R to carry out two multiple regression analyses.

**Fig 6:**
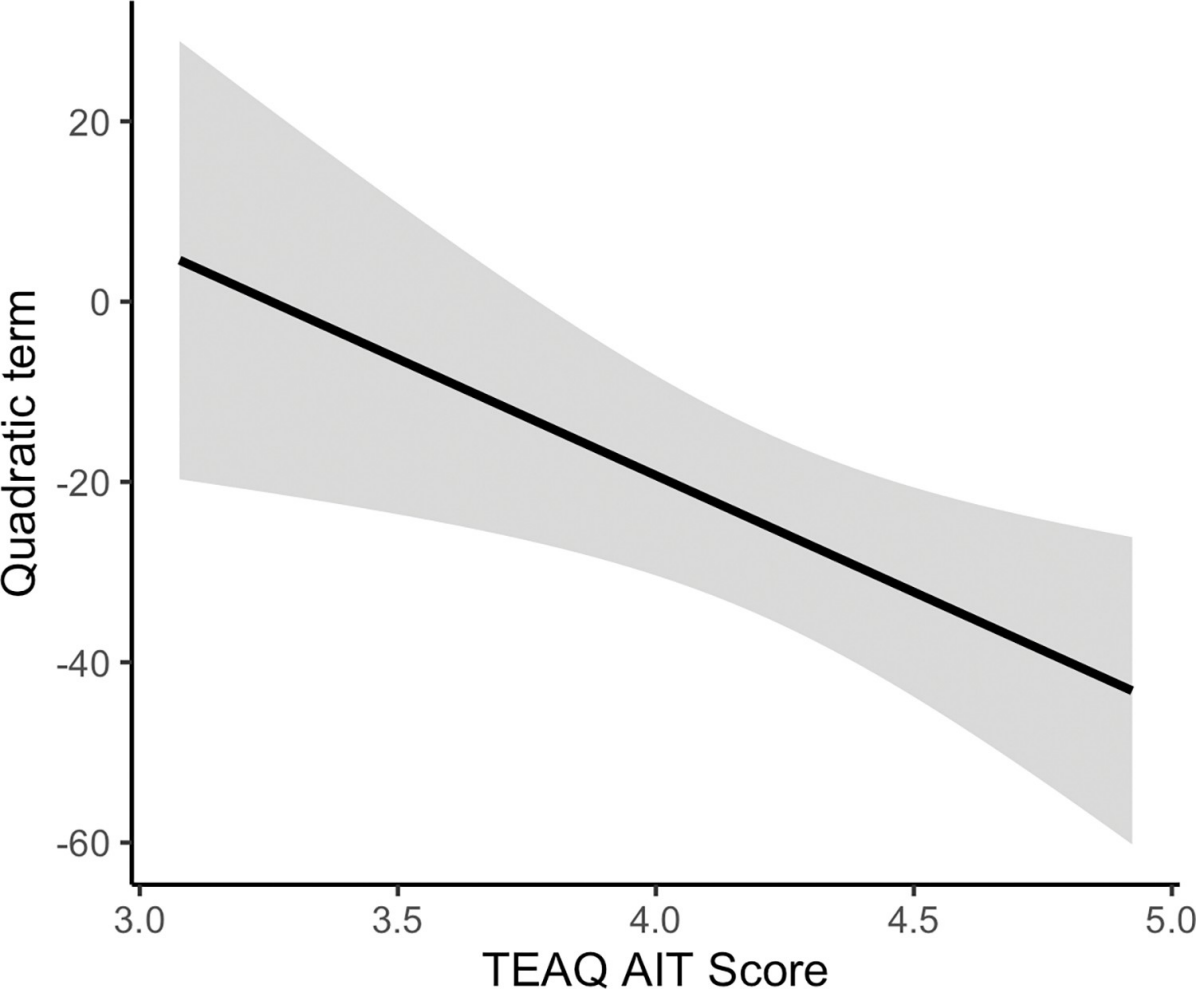
Relationship between attitude to intimate touch (AIT, as determined by the Touch Experiences and Attitudes Questionnaire (TEAQ)) and the quadratic velocity terms for 0.4 N touch. The linear regression line with 95% confidence interval band is shown. More positive attitudes towards intimate touch are related to a more negative quadratic term and therefore steeper inverted U-shaped relationship between touch pleasantness and stroking velocity, indicating greater sensitivity towards CT-targeted touch (*p* = .014).

*TEAQ subscales as predictors of participants’ quadratic terms for 0.4 N force.* A multiple regression analysis with the outcome variable as the quadratic velocity terms for the 0.4 N force and the predictors as the six TEAQ subscales, identified the TEAQ subscales to significantly predict the 0.4 N quadratic velocity terms, explaining 27% of the variance (*R*^*2*^ = .42, adjusted *R*^2^ = .27, *F*(6, 23) = 2.78, *p* = .035). It was identified that the attitude to intimate touch (AIT) subscale significantly predicted the quadratic velocity terms for 0.4 N (*F*(1, 23) = 7.14, *p* = .014). A negative relationship was identified, with a more negative quadratic term, and therefore a steeper inverted U, which indicates greater sensitivity towards CT-targeted touch, being related to more positive attitudes to intimate touch. This relationship is presented in Fig 6.

The current intimate touch (CIT) scale was identified as a marginally significant predictor of the quadratic velocity terms for 0.4 N touch (*F*(1, 23) = 3.68, *p* = .067). A positive relationship was identified, where a more negative quadratic term and therefore steeper inverted U was related to lower levels of current intimate touch. The attitude to unfamiliar touch (AUT) scale was also marginally significantly predictive of the quadratic velocity term for 0.4 N touch (*F*(1, 23) = 3.60, *p* = .071). More negative attitudes to unfamiliar touch were related to a more negative quadratic term and therefore a steeper inverted U. Friends and family touch (FFT), childhood touch (ChT) and attitude to self-care (AtSC) were not significantly predictive of the quadratic term for 0.4 N touch (*Fs*(1, 23) ≤ 2.40, *p*s ≥ .135). These findings indicate that attitudes towards and experiences of intimate touch are predictive of a person’s sensitivity to the specific rewarding value of CT-optimal affective touch.

*Investigating stress, depression and autistic traits as predictors of the quadratic velocity terms for 0.4 N touch.* A multiple regression analysis was carried out to determine whether the total score for the autism quotient (AQ), the total score for the perceived stress scale (PSS) and the measure of depressive symptoms determined by the Patient Health Questionnaire (PHQ-9), were significantly predictive of the quadratic velocity terms for 0.4 N touch. It was identified that the three measures combined did not explain a significant amount of the variance in the quadratic velocity terms for 0.4 N touch (*R*^2^ = .02, adjusted *R*^2^ = -.09, *F*(3, 25) = 0.197, *p* = .898). Thus neither current perceived stress, depressive symptomology or autistic traits accounted for a significant proportion of the variance in the quadratic velocity terms for 0.4 N touch.

*Predictors of static robotic touch ratings*. It was investigated whether there was a significant effect of force on static robotic touch ratings. No significant effect of force was identified (*F*(2, 234.102) = 0.61, *p* = .546). As previous literature and the results of this analysis do not implicate an optimal force in terms of pleasantness ratings of static touch, all participant data was included in the analysis of static touch ratings, regardless of force applied. Two mixed effects models were therefore used, with a random effect of participant, to investigate whether the individual differences measures included in the study significantly predicted pleasantness ratings for static robotic touch.

*TEAQ subscales as predictors of static robotic touch pleasantness ratings*. A mixed effects model was used to determine if any of the six TEAQ subscales were significantly predictive of pleasantness ratings of static robotic touch. It was identified that none of the subscales were significantly predictive (*F*s ≤ 2.28, *p*s ≥ 0.144).

**Fig 7:**
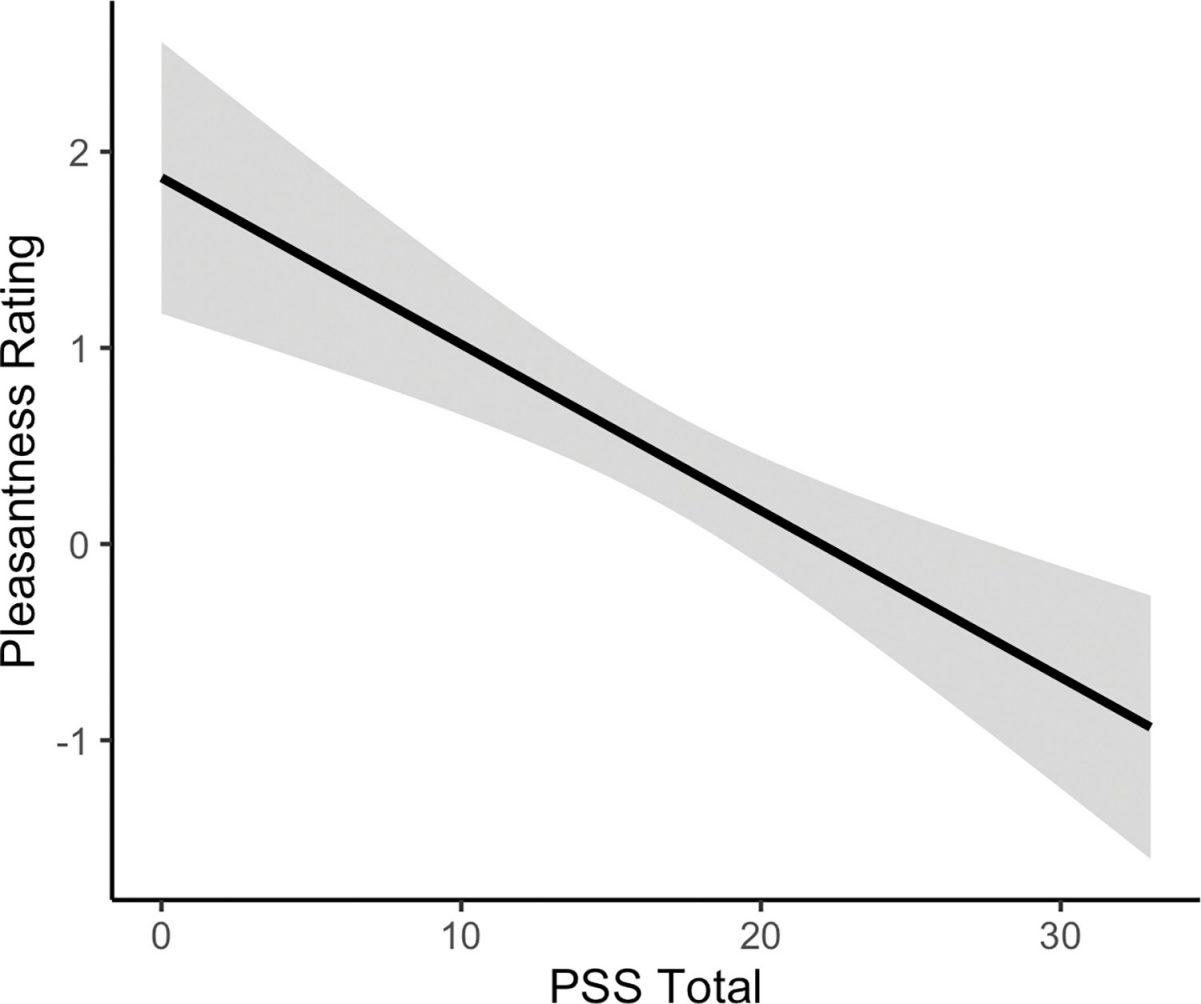
Relationship between perceived stress (as measured by the Perceived Stress Scale (PSS)) and static robotic touch pleasantness ratings. The linear regression line with 95% confidence interval band is shown. Greater perceived stress was related to reduced static robotic touch pleasantness (*p* = .002).

*Investigating stress, depression and autistic traits as predictors of static touch pleasantness ratings*. A mixed effects model was used to determine if AQ total score, PSS score and PHQ-9 score were significantly related to pleasantness ratings of static robotic touch. Perceived stress during the last month (PSS score) was significantly negatively related to ratings of static robotic touch pleasantness, with increasing stress levels related to decreasing pleasantness ratings of static touch (*F*(1, 26.074) = 12.40, *p* = .002). This relationship is presented in Fig 7. Autism quotient (AQ) total score and depressive symptoms, as measured by the PHQ-9, were not significantly related to ratings of static robotic touch pleasantness (*F*s ≤ 2.82, *p*s ≥ .105).

*Individual differences analysis for videos data*. This analysis was carried out on all participant data combined (lab and online data). The quadratic velocity term was calculated for each participant separately for each location and question for dynamic touch only. The static touch data was removed from this analysis. Log_10_ velocity was used, as the velocities selected for this study were chosen based on their equidistance on a log scale.

To determine whether there was a significant effect of question and location on the quadratic velocity term, a mixed effects model was used with the quadratic term as the dependent variable, question and location as fixed effects and a random effect of participant. The interaction between question and location was not significant (*F*(4, 2275.0) = 0.30, *p* = .875) and no significant effect of question was identified (*F*(1, 2275.0) = 0.35, *p* = .552).

A significant effect of location was identified (*F*(4, 2275.2) = 8.22, *p* < .001). Estimated marginal means comparison with FDR correction for multiple comparisons identified the quadratic velocity term for the dorsal forearm to be significantly more negative, representing a steeper inverted U, than for all other body sites (*t*s ≥ 3.82, *p*s < .001). The differences between the quadratic terms for all other locations were not significant (*t*s ≤ 1.50, *p*s ≥ .268).

As no effect of question was identified, but an effect of location was identified, a quadratic term averaged over question was calculated for each participant. As the quadratic term was steepest for the dorsal forearm, the analysis was carried out on the dorsal forearm data only.

**Fig 8:**
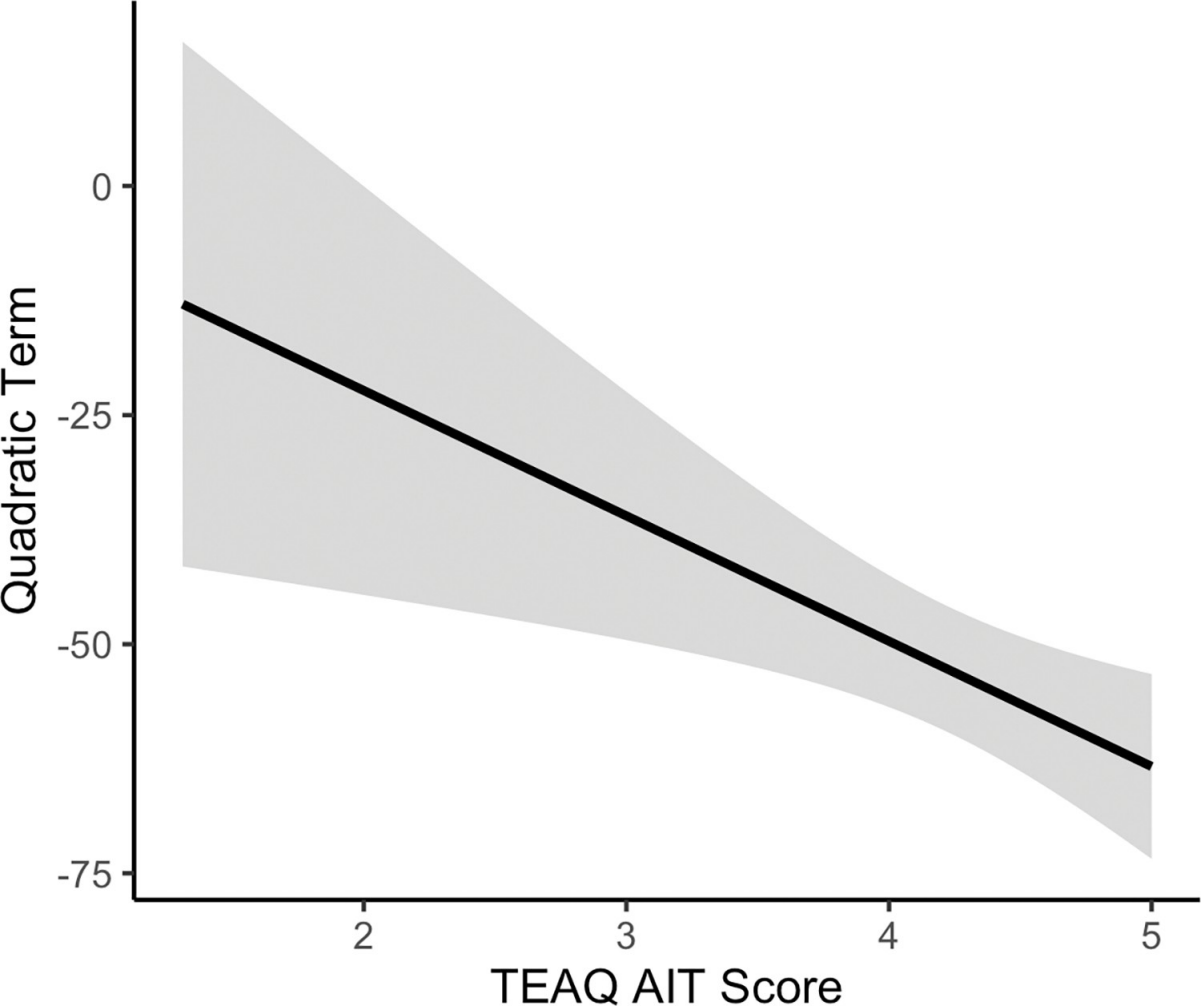
Relationship between attitude to intimate touch (AIT, as determined by the Touch Experiences and Attitudes Questionnaire (TEAQ)) and the quadratic velocity terms for vicarious dorsal forearm touch. The linear regression line with 95% confidence interval band is shown. More positive attitudes towards intimate touch are related to a more negative quadratic term and therefore a steeper inverted U-shaped relationship between touch pleasantness and stroking velocity, indicating greater sensitivity towards CT-targeted touch (*p* = .031).

*TEAQ subscales as predictors of individual quadratic terms for vicarious ratings of dynamic dorsal forearm touch*. A multiple regression analysis was used to determine whether the six TEAQ subscales were predictive of the quadratic velocity term calculated for each participant for the dorsal forearm collapsed over question. The six TEAQ subscales were entered as predictors. The TEAQ subscales were identified to significantly predict the dorsal forearm quadratic velocity terms, explaining 3.3% of the variance (*R*^*2*^ = .06, adjusted *R*^2^ = .03, *F*(6, 244) = 2.41, *p* = .028). It was identified that the attitude to intimate touch (AIT) subscale significantly predicted the quadratic velocity term for dorsal forearm touch (*F*(1, 244) = 4.71, *p* = .031). A negative relationship was identified, with a more negative quadratic term, and therefore a steeper inverted U, which indicates greater sensitivity towards CT-targeted touch, being related to more positive attitudes towards intimate touch. This relationship is presented in Fig 8. None of the other TEAQ subscales were significantly predictive of the quadratic velocity term (*F*s(1, 244) ≤ 2.27, *p*s ≥ .133). This result is consistent with the results of the robotic touch ratings analysis, indicating that positive attitudes towards intimate touch are related to greater sensitivity towards CT targeted vicarious touch.

*Investigating stress, depression and autistic traits as predictors of individual quadratic terms for vicarious dorsal forearm touch*. A multiple regression analysis was used to determine whether PSS score, AQ total score and PHQ-9 score predicted the individual quadratic velocity terms for dorsal forearm vicarious touch collapsed over question. The three predictors combined did not explain a significant amount of the variance in the quadratic velocity terms (*R*^*2*^ = 0.006, adjusted *R*^*2*^ = -0.006, *F*(3, 246) = 0.49, *p* = .691). Thus, consistent with the robotic touch findings, current perceived stress, depressive symptomology, and autistic traits do not account for a significant proportion of the variance in the quadratic velocity terms for vicarious dorsal forearm touch.

*Ratings of static touch*. This analysis was carried out on all participant data combined (lab and online data). To determine whether there was a significant effect of question and location on static touch ratings, a mixed effects model was used with ratings of static touch as the dependent variable, question and location as fixed effects and a random effect of participant. The interaction between question and location was not significant (*F*(4, 2277) = 0.74, *p* = .568). However, the main effect of question was significant (*F*(1, 2277) = 86.56, *p* < .001). Ratings for how pleasant the touch was for the person in the video (other-directed touch) were significantly higher than ratings for when participants were asked how much they would like to be touched like that (self-directed touch).

Additionally, there was a significant main effect of location (*F*(4, 2277) = 88.31, *p* < .001). Ratings were significantly greater for the dorsal hand than any other location (*t*s(2277) ≥ 9.90, *p*s < .001).

**Fig 9:**
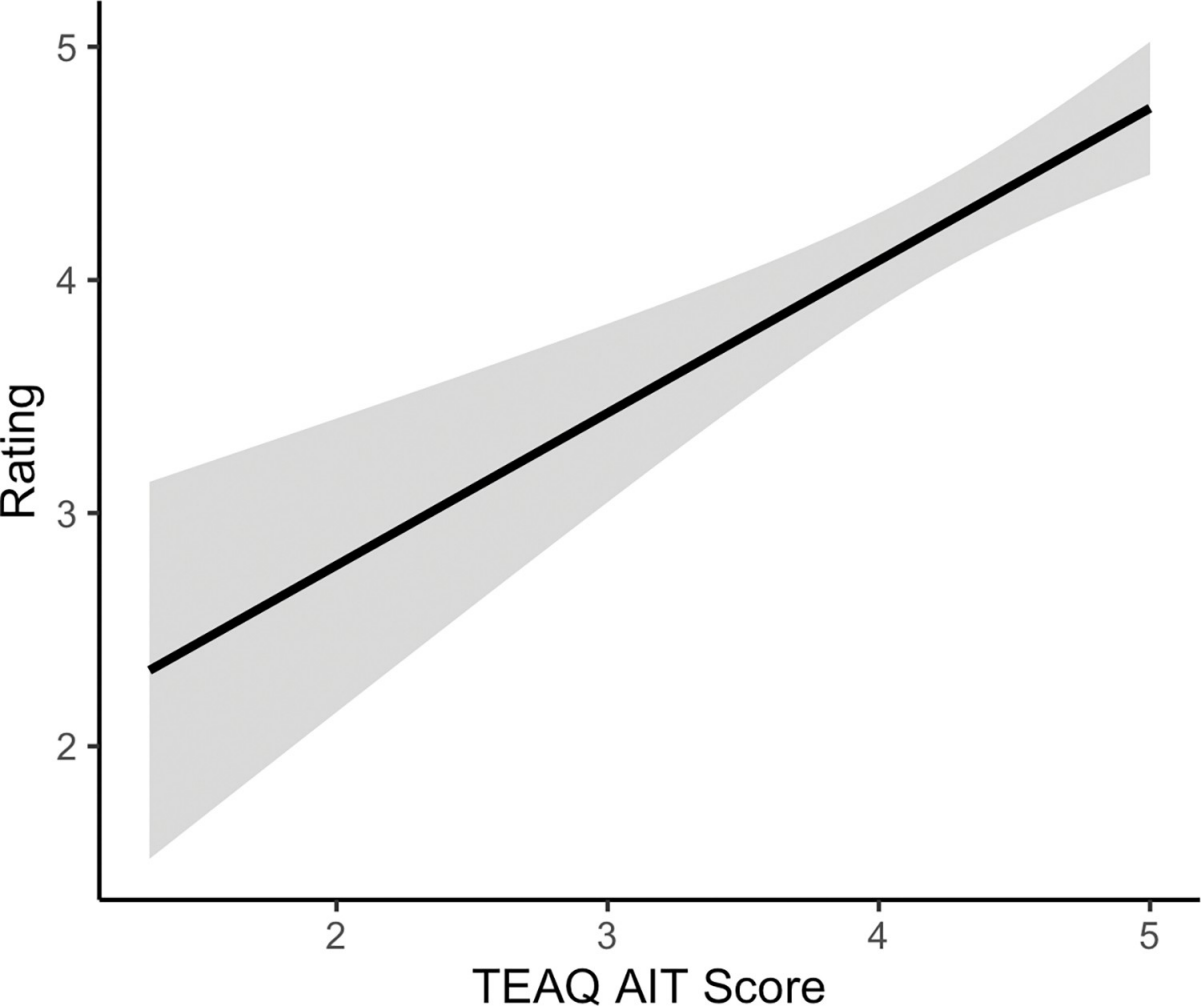
Relationship between attitude to intimate touch (AIT, as measured by the Touch Experiences and Attitudes Questionnaire (TEAQ)) and vicarious, self-focussed, dorsal hand touch ratings. The linear regression line with 95% confidence interval band is shown. More positive attitudes to intimate touch are related to more positive static touch ratings (*p* = .016).

*TEAQ subscales as predictors of static touch ratings*. As location and question were identified to have a significant effect on static touch ratings, it was considered important to investigate whether ratings of static touch applied to the dorsal hand, which was on average rated most positively, were significantly predicted by the six TEAQ subscales using a multiple regression analysis. The analysis was run for the self-focussed question responses, as this question was deemed more directly relevant than the other-focussed question. Overall, the six TEAQ subscales explained a significant amount of the variance in static touch ratings for the dorsal hand, self-focussed question, explaining 7.6% of the variance (*F*(6, 244) = 4.43, *p* < .001). The attitude to intimate touch (AIT) subscale was significantly positively predictive of static touch ratings (*F*(1, 244) = 5.92, *p* = .016). More positive attitudes to intimate touch were predictive of more positive ratings of static touch. This relationship is presented in Fig 9. None of the other TEAQ subscales were significant predictors of static touch ratings (*F*s(1, 244) ≤ 2.43, *p* = .120).

This analysis provides partial support for our hypothesis that positive attitudes and experiences of touch would be related to more positive ratings of vicarious touch. Specifically, attitudes to intimate touch are positively related to vicarious ratings of static touch to the dorsal hand. These results are not consistent with those obtained for robotic touch, as the TEAQ subscales were identified to not significantly predict static robotic touch ratings.

*Investigating stress, depression and autistic traits as predictors of static touch ratings*. A multiple regression analysis was used to determine whether AQ total score, PSS score and PHQ-9 score were significantly predictive of vicarious static touch ratings for the dorsal hand in response to the self-focussed question. Overall, the three measures did not predict a significant amount of the variance in static touch ratings (*R*^*2*^ = 0.008, adjusted *R*^*2*^ = -0.004, *F*(3, 246) = 0.68, *p* = .568). This is not consistent with the robotic touch data, where PSS scores were found to negatively predict static touch ratings.

## Discussion

This study aimed to broaden our understanding of the social touch hypothesis by investigating the relative preference for static vs dynamic touch and the influence of force on these preferences. Additionally, we aimed to determine whether self-reported attitudes toward and experiences of affective touch, autistic traits, perceived stress, and depressive symptomology, could account for some of the individual differences in affective touch responses previously reported.

Our results provided support for our hypothesis that there would be a preference for static compared to CT non-optimal touch, but did not support our second hypothesis that static touch may be preferred at a higher force (1.5 N) than dynamic touch. Our hypothesis that positive attitudes toward and experiences of affective touch would be related to more positive psychophysical and vicarious ratings of touch and greater sensitivity to CT targeted touch was generally supported. Finally, our study provided evidence to support the hypothesis that stress would be associated with reduced affective touch pleasantness, however, depressive symptomology and autistic traits were not significantly associated with touch pleasantness.

In terms of preference for static compared to CT non-optimal dynamic touch, ratings of static robotic touch pleasantness were identified as significantly more pleasant than 0.3 cm/s robotic touch, but comparable to 30 cm/s robotic touch applied to the ventral forearm. For vicarious touch ratings, overall, static touch was rated more positively than both CT non-optimal velocities investigated (0.5 and 30 cm/s). These results therefore support the hypothesis that static touch is generally preferred over CT-non-optimal, but not CT-optimal dynamic touch. This is supported by the literature, in that CT firing frequency strongly correlates with touch pleasantness and CT firing frequency is optimal for stroking touch applied at 1–10 cm/s, with lower firing frequency and therefore lower pleasantness ratings at slower and faster velocities [1]. There is also evidence that CTs are activated by static as well as dynamic touch [8, 15, 18], thus these results may be explained by the variation in CT’s firing frequency produced by touches of differing velocities. Investigating the firing frequency of CTs to static compared to CT optimal and non-optimal dynamic touch has yet to be carried out, so whether or not these results are directly related to CT firing frequency is currently unknown.

When considering the effect of velocity, the pattern of results for CT non-optimal stroking touch was not the same for robotic vs vicarious touch. While CT optimal touch was rated as most pleasant for both touch modalities, viewing touch applied to the ventral forearm resulted in 30 cm/s touch being rated as significantly less pleasant than 0.5 cm/s touch. However, for robotic touch applied to the ventral forearm, 0.3 cm/s touch was rated as least pleasant and significantly less pleasant than 30 cm/s touch. This may in part be due to methodological differences. All of our touch videos had the same duration (6 s), however for robotic touch, one stroke was delivered per trial, which means the duration of the slowest velocity (0.3 cm/s) stoke was ~ 15 s, compared to ~ 0.15 s for a 30 cm/s stroke. The long stroking duration for 0.3 cm/s robotic touch is likely to have made this touch rather boring and irritating, whereas viewing a very slow stroke lasting less than half this time appears to have been perceived as more tolerable for our participants. We also have to bear in mind the lack of peripheral input for vicarious touch. While previous literature has identified the posterior insula to be activated by both vicarious and directly experienced touch [3, 53], it has to be accepted that these touch modalities are not directly comparable in terms of peripheral input and therefore central responses. Additionally, the touch videos depicted interpersonal, rather than robotic touch. For future studies, if direct comparisons between responses to directly experienced and vicarious touch are required, it should be ensured that stimulus durations are the same and that the videos depict the same type of touch as the directly experienced touch.

Our results highlight the context-dependent nature of affective touch responses in that central, as well as peripheral encoding needs to be considered. Our vicarious touch responses identified a location by velocity interaction, highlighting relative preference for static compared to dynamic touch was dependent on body location. In particular, ratings for touch to the dorsal hand, where static touch was rated equally pleasant as CT optimal, 3 cm/s touch. CT innervation density has been identified to be similar throughout the forearm and dorsal hand in humans [54]. It is therefore unlikely this result is due to differences in CT innervation density. A more likely explanation is the social relevance of static touch to the hand, with this region associated with static hand-holding, more so than stroking touch [55]. That this result was seen for touch to the dorsal hand, but not the palm suggests a potential CT contribution, due to greater CT innervation of hairy compared to glabrous skin [56]. This may be due to the thermal sensitivity of CTs to human skin temperature [2], rather than the velocity tuning of CTs.

For all other body sites investigated, except the ventral forearm, vicarious static touch was rated as significantly less pleasant than CT optimal touch, but more pleasant than CT non-optimal dynamic touch, potentially due to this static touch of a hand being applied to the arm or palm being more socially acceptable and more commonly encountered than very slow or fast dynamic touch. Ratings comparing social acceptability and tolerance of different touch velocities have not been obtained previously and are aspects which could be considered for future investigations. It is unclear why touch to the ventral forearm led to a different pattern of results, with 0.3 cm/s touch rated as significantly more pleasant than static and 30 cm/s touch, with comparable ratings for static and 30 cm/s touch, particularly as this does not reflect the ratings obtained for robotic touch. The ecological validity of the stimuli used in this study needs to be considered, as the videos used were highly controlled and accurate in terms of stroking velocities, but do not necessarily reflect naturally occurring social gestures in real-world settings. This is also the case for the robotic touches delivered. It may well be the case that a different pattern of results would be obtained with more naturalistic, ecologically valid touch stimuli, e.g. videos showing gestures such as hugs and handholding.

In terms of the effect of force on touch ratings, we hypothesised that static touch would be preferred at a higher force (1.5 N) than dynamic touch, however, our results did not support this hypothesis. We identified no significant interaction effect of force with velocity, instead identifying the force of 0.4 N typically used in affective touch research [e.g. 1, 2, 57, 58] was preferred over lower and higher force touch. This supports previous literature that touch applied at a force of 0.4 N is typically associated with a pleasant percept [e.g. 1, 58, 59]. Whether or not this reflects force, as well as velocity tuning of CTs, with optimal CT activation occurring at a force of around 0.4 N remains to be identified. As discussed in the introduction, evidence suggests this may not be the case, as greater CT activation with increasing force up to a force of 0.1 N has been identified [12–14]. However, evidence suggests CTs may respond similarly to forces greater than this [1, 8, 15], suggesting top-down influences may also contribute to this effect. Thus, further investigation into the effect of force on CT activation is required.

Our hypothesis that static touch would be preferred at higher forces was proposed based on the observation that socially relevant static touch, such as hugging and holding touches, may be typically associated with greater forces than caressing touch, although this has yet to be measured objectively. However, as the touches rated in the present study were delivered via the RTS, they did not optimally replicate these types of touch. An investigation obtaining ratings for naturally occurring static touches, as well as measuring the physical properties of these touches would be of value. Alternatively, our results may be due to deep pressure touch being encoded by a pathway other than the CT pathway [23], leading to lower pleasantness ratings due to physiological differences in the encoding of high compared to low force touch.

When considering affective touch responses and the contribution of central and peripheral encoding, as well as the context-dependent nature of affective touch, we also need to consider the influence of individual differences on these responses. A recent report identified that despite there being a well replicated inverted-U relationship between affective touch pleasantness and velocity when looking at grouped data, individual responses to affective touch show variation in this relationship, with some participants’ ratings displaying a much steeper inverted-U relationship than others [24]. The current study therefore aimed to investigate this further by determining if measures of key individual differences previously identified as influencing affective touch responses, could explain some of the variability in this inverted-U relationship between touch pleasantness and velocity. As hypothesised, we identified that positive attitudes toward and experiences of affective touch were associated with greater sensitivity to CT targeted touch. Specifically, attitudes toward and experiences of affective touch, as measured by the six subscales of the Touch Experiences and Attitudes Questionnaire (TEAQ) [25], explained a significant amount of the variance in participants’ quadratic velocity terms (i.e. the steepness of their inverted U relationship between touch pleasantness and dynamic touch velocity) for 0.4 N robotic dynamic touch and vicarious dynamic dorsal forearm touch. Interestingly, of the six TEAQ subscales, only the attitude to intimate touch subscale was significantly predictive of participants’ quadratic velocity terms, with more positive attitudes towards intimate touch being associated with a steeper inverted-U relationship and therefore greater sensitivity towards CT-targeted touch. Within the TEAQ, only the two intimate touch subscales, measuring attitudes towards (AIT) and levels of intimate touch currently experienced (CIT), contain items specifically relating to stroking and therefore CT-targeted touch. This may well explain why the AIT subscale appears specifically related to sensitivity to CT-targeted touch. It suggests that there may be individual differences in sensitivity of the CT system and that these differences relate psychologically to a more positive attitude towards intimate touch. That the current intimate touch measure was not significantly predictive suggests that this sensitivity towards CT optimal touch is not influenced by current level of CT stimulation, but that sensitivity towards CT optimal touch may well be relatively stable over time. This is supported by the results of Sehlstedt *et al*., [60] who identified no significant difference in the inverted-U relationship between stroking velocity and perceived touch pleasantness when comparing ratings of adolescents (aged 13–18), younger adults aged 19–44 and older adults aged 45–82, however, they identified an increase in overall touch pleasantness with increasing age.

Contrary to our hypothesis, the six TEAQ subscales were not significantly predictive of ventral forearm robotic static touch ratings, however, they were for self-focussed vicarious ratings of static touch applied to the dorsal hand. This may again be in part due to the ecological validity of these two types of touch, with static touch applied to the dorsal hand by another individual’s hand being closer to the social context in which this type of touch may be received than robotic static touch applied to the ventral forearm, a body region less commonly associated with static, such as holding, touch [55]. Again, the attitude to intimate touch subscale was the only subscale which was significantly predictive of self-focussed vicarious ratings of static touch applied to the dorsal hand. This is interesting, as questions relating to attitudes towards static touch can be identified in the friends and family touch (FFT) subscale and attitude to unfamiliar touch (AUT) subscale, as well as the attitude to intimate touch (AIT) subscale. However, the static touch items in the AIT subscale relate to more prolonged static touches, including hand holding and snuggling up on the sofa with someone, whereas the touches referred to in the FFT subscale generally relate to briefer static touches, such as hugging when greeting someone. The AUT subscale refers to touch generally from people the participant does not know very well, so would also suggest briefer touch durations. As discussed in the introduction, CTs have been reported to be intermediate adapting and thus show sustained firing during static touch [15, 17, 18],. Additionally, considering the temperature tuning of CTs [2], prolonged touch may lead to greater CT activation than briefer touches, due to the presence of a warm stimulus for a longer duration. Therefore, the static touches relating to the attitude to intimate touch subscale may relate to greater CT activation than the touches associated with the other subscales, explaining why this type of touch is predictive of pleasantness ratings of static touch. Measuring CT responses to socially relevant brief and prolonged static touches would further our understanding of CT physiology, as well as helping to explain our social touch behaviours.

In terms of individual differences, we also investigated the influence of depression, stress and autistic traits on robotic and vicarious touch responses. We hypothesised stress, depressive symptomology, and autistic traits would be associated with reduced affective touch pleasantness. However, we obtained little evidence to support this hypothesis. These scales did not significantly predict the quadratic terms for robotic or vicarious dynamic touch responses. Further they did not predict pleasantness ratings of self-focussed, vicarious ratings of static touch applied to the dorsal hand. However, current levels of perceived stress were identified to negatively predict pleasantness ratings of static, robotic touch applied to the ventral forearm. Higher levels of perceived stress were associated with lower ratings of static touch pleasantness. This may be due to directly experienced touch having a direct, physiological effect, the bottom-up encoding of which is likely influenced by the effect of chronic stress on reducing dopamine transmission in the mesolimbic pathway [32] more so than vicarious touch responses, for which there are likely more top-down influences.

The analysis of the effect of individual differences on sensitivity towards CT optimal touch, as indicated by individual’s quadratic terms, suggests this sensitivity is relatively stable over time, seemingly not influenced by levels of depression and stress, as well as not being influenced by levels of affective touch currently experienced. This is in line with previous literature which identified no influence of depression on affective touch awareness [39]. That levels of autistic traits did not predict sensitivity towards CT optimal touch or static touch responses is perhaps more surprising, given previous literature has identified altered affective touch responses in autistic individuals and those with high levels of autistic traits [37–40, 61, 62], although many of these studies have identified evidence of neuronal, rather than perceptual differences. A limitation of this study was that the current sample was not a clinical sample, therefore the levels of autistic traits were relatively low, with only one participant for the laboratory study and 2.8% of the whole sample having an Autism Quotient score of 32 or greater, a score highly predictive of autism [41]. Further investigation into vicarious and robotic static and dynamic touch responses of participants with and without a diagnosis of an autism spectrum condition would further our understanding of how autistic traits influence affective touch responses.

In addition to the limitations and future directions already identified, some more general limitations should also be noted. The sample included in this study was predominantly female and for the online version was relatively young. The results may therefore not be representative of the general wider population. Sex differences in affective touch responses have been previously identified [63], therefore the current study may not be representative of male affective touch responses. As recruitment was limited to the North West of England, this study does not account for cultural variation in affective touch responses.

To further this investigation, obtaining responses to directly experienced and vicarious static vs dynamic touch for more ecologically valid touch stimuli, such as hugging, holding, and stroking touch between romantic partners and between parents and their children, rather than the highly controlled, but less socially relevant stimuli used in this study, would provide a more naturalistic investigation of static vs dynamic touch. Crucially, further microneurographic recordings of CT touch responses to static vs dynamic touch would allow us to understand the contribution of CTs in the encoding of static touch. This could be further addressed using brain imaging techniques, such as fMRI to see if the pattern of brain activation is similar for static vs dynamic touch, or whether it is different, suggesting a different neural pathway for static touch, as suggested for deep pressure touch [23]. Further, a focus on implicit and biochemical, as well as explicit responses to touch using electrophysiology and biochemical assays of cortisol and oxytocin, would provide greater insight into the physiological benefits of static vs dynamic touch, in terms of reducing physiological arousal, cortisol reactivity and increasing oxytocin levels, as already implicated for dynamic, CT-targeted touch [64–71].

In conclusion, this study has identified that individual differences in sensitivity to CT targeted touch can be predicted by attitudes to intimate touch, but not by levels of depression, stress, or autistic traits. In general, we also identified static touch to be preferred over CT-non optimal, but not CT-optimal dynamic touch, suggesting stroking to be preferred over holding touch, but only if the stroking touch is delivered at a medium velocity (between 1–10 cm/s). The context dependent nature of affective touch responses was highlighted by the differing pattern of responses obtained for touches applied to different body sites. Overall, this study has highlighted the need to consider individual differences and the relative importance of static, as well as dynamic touch when investigating affective touch responses.

## Supporting information

S1 File Results of the intensity data analysis have been provided as supplementary materials. This file contains the results of this analysis, as well as the figures depicting the effect of force and velocity on intensity ratings.
(PDF)

## References

[1] Löken LS, Wessberg J, Morrison I, McGlone F, Olausson H. Coding of pleasant touch by unmyelinated afferents in humans. Nat Neurosci. 2009;12(5):547–8. doi: 10.1038/nn.2312 19363489

[2] Ackerley R, Wasling HB, Liljencrantz J, Olausson H, Johnson RD, Wessberg J. Human C-tactile afferents are tuned to the temperature of a skin-stroking caress. J Neurosci. 2014;34(8):2879–83. doi: 10.1523/JNEUROSCI.2847-13.2014 24553929 PMC3931502

[3] Olausson HW, Lamarre Y, Backlund H, Morin C, Starck G, Ekholm S, et al. Unmyelinated tactile afferents signal touch and project to the insular cortex. Nat Neurosci. 2002;5:900–4. 12145636 10.1038/nn896

[4] McGlone F, Wessberg J, Olausson H. Discriminative and Affective Touch: Sensing and Feeling. Neuron. 2014;82:737–55. doi: 10.1016/j.neuron.2014.05.001 24853935

[5] Gordon I, Voos AC, Bennett RH, Bolling DZ, Pelphrey KA, Kaiser MD. Brain mechanisms for processing affective touch. Hum Brain Mapp. 2013;34(4):914–22. doi: 10.1002/hbm.21480 22125232 PMC6869848

[6] Mcglone F, Olausson H, Boyle JA, Jones-Gotman M, Dancer C, Guest S, et al. Touching and feeling: Differences in pleasant touch processing between glabrous and hairy skin in humans. Eur J Neurosci. 2012;35(11):1782–8. doi: 10.1111/j.1460-9568.2012.08092.x 22594914

[7] Touch Zotterman Y., pain and tickling: an electro‐physiological investigation on cutaneous sensory nerves. J Physiol. 1939;95(1):1–28. 16995068 10.1113/jphysiol.1939.sp003707 PMC1393960

[8] Nordin M. Low‐threshold mechanoreceptive and nociceptive units with unmyelinated (C) fibres in the human supraorbital nerve. J Physiol. 1990;426(1):229–40. doi: 10.1113/jphysiol.1990.sp018135 2231398 PMC1189885

[9] Vallbo Å, Olausson H, Wessberg J, Norrsell U. A system of unmyelinated afferents for innocuous mechanoreception in the human skin. Brain Res. 1993;628(1–2):301–4. doi: 10.1016/0006-8993(93)90968-s 8313159

[10] Morrison I, Löken LS, Olausson H. The skin as a social organ. Exp Brain Res. 2010;204(3):305–14. doi: 10.1007/s00221-009-2007-y 19771420

[11] Olausson HW, Wessberg J, Morrison I, McGlone F, Vallbo A. The neurophysiology of unmyelinated tactile afferents. Neurosci Biobehav Rev. 2008/10/28. 2010;34(2):185–91. doi: 10.1016/j.neubiorev.2008.09.011 18952123

[12] Middleton SJ, Perini I, Themistocleous AC, Weir GA, McCann K, Barry AM, et al. Nav1. 7 is required for normal C-low threshold mechanoreceptor function in humans and mice. Brain [Internet]. 2021; Available from: 10.1093/brain/awab482 PMC9586547 34957475

[13] Wessberg J, Olausson H, Fernstrom KW, Vallbo AB. Receptive field properties of unmyelinated tactile afferents in the human skin. J Neurophysiol. 2003;89(3):1567–75. doi: 10.1152/jn.00256.2002 12626628

[14] Ackerley R. C-tactile (CT) afferents: evidence of their function from microneurography studies in humans. Curr Opin Behav Sci. 2022;

[15] Vallbo AB, Olausson H, Wessberg J. Unmyelinated afferents constitute a second system coding tactile stimuli of the human hairy skin. J Neurophysiol. 1999;81(6):2753–63. doi: 10.1152/jn.1999.81.6.2753 10368395

[16] Gilbert C, McCafferty D, Le Maho Y, Martrette JM, Giroud S, Blanc S, et al. One for all and all for one: The energetic benefits of huddling in endotherms. Biol Rev. 2010;85(3):545–69. doi: 10.1111/j.1469-185X.2009.00115.x 20039866

[17] Ackerley R, Watkins RH. Microneurography as a tool to study the function of individual C-fiber afferents in humans: Responses from nociceptors, thermoreceptors, and mechanoreceptors. Journal of Neurophysiology. 2018. 10.1152/jn.00109.2018 30256737

[18] Ackerley R, Fernström KW, Wasling HB, Watkins RH, Johnson RD, Vallbo Å, et al. Differential effects of radiant and mechanically applied thermal stimuli on human C-tactile afferent firing patterns. J Neurophysiol. 2018; doi: 10.1152/jn.00940.2017 30044679

[19] Feldman R, Eidelman AI. Skin-to-skin contact (Kangaroo Care) accelerates autonomic and neurobehavioural maturation in preterm infants. Dev Med Child Neurol. 2003;45(4):274–81. doi: 10.1017/s0012162203000525 12647930

[20] Cunningham C, Moore Z, Patton D, O’Connor T, Nugent LE. Does Kangaroo care affect the weight of preterm/low birth-weight infants in the neonatal setting of a hospital environment? J Neonatal Nurs. 2018 Aug;24(4):189–95.

[21] Acolet D, Sleath K, Whitelaw A. Oxygenation, heart rate and temperature in very low birthweight infants during skin-to-skin contact with their mothers. Acta Paediatr Scand. 1989 Mar;78(2):189–93. doi: 10.1111/j.1651-2227.1989.tb11055.x 2929342

[22] Cong X, Cusson RM, Walsh S, Hussain N, Ludington-Hoe SM, Zhang D. Effects of Skin-to-Skin Contact on Autonomic Pain Responses in Preterm Infants. J Pain. 2012 Jul;13(7):636–45. doi: 10.1016/j.jpain.2012.02.008 22595172

[23] Case LK, Liljencrantz J, McCall M V., Bradson M, Necaise A, Tubbs J, et al. Pleasant Deep Pressure: Expanding the Social Touch Hypothesis. Neuroscience. 2021; doi: 10.1016/j.neuroscience.2020.07.050 32768616 PMC7865002

[24] Croy I, Bierling A, Sailer U, Ackerley R. Individual Variability of Pleasantness Ratings to Stroking Touch Over Different Velocities. Neuroscience. 2021; doi: 10.1016/j.neuroscience.2020.03.030 32224227

[25] Trotter PD, McGlone F, Reniers RLEP, Deakin JFW. Construction and Validation of the Touch Experiences and Attitudes Questionnaire (TEAQ): A Self-report Measure to Determine Attitudes Toward and Experiences of Positive Touch. J Nonverbal Behav. 2018;42:379–416. doi: 10.1007/s10919-018-0281-8 30416240 PMC6208655

[26] Devine SL, Walker SC, Makdani A, Stockton ER, McFarquhar MJ, McGlone FP, et al. Childhood Adversity and Affective Touch Perception: A Comparison of United Kingdom Care Leavers and Non-care Leavers. Front Psychol. 2020; doi: 10.3389/fpsyg.2020.557171 33240148 PMC7683385

[27] Trotter PD, McGlone F, McKie S, McFarquhar M, Elliott R, Walker SC, et al. Effects of acute tryptophan depletion on central processing of CT-targeted and discriminatory touch in humans. Eur J Neurosci. 2016;44(4):2072–83. doi: 10.1111/ejn.13298 27307373

[28] Triscoli C, Croy I, Sailer U. Depression predicts interpersonal problems partially through the attitude towards social touch. J Affect Disord. 2019; doi: 10.1016/j.jad.2018.12.054 30584957

[29] Hernandez-Reif M, Field T, Krasnegor J, Theakston H. Lower back pain is reduced and range of motion increased after massage therapy. Int J Neurosci. 2001;106:131–45. doi: 10.3109/00207450109149744 11264915

[30] Morrison I. Keep Calm and Cuddle on: Social Touch as a Stress Buffer. Adapt Hum Behav Physiol. 2016;2(4):344–62.

[31] Von Mohr M, Kirsch LP, Fotopoulou A. The soothing function of touch: Affective touch reduces feelings of social exclusion. Sci Rep. 2017;7(1):13516. doi: 10.1038/s41598-017-13355-7 29044137 PMC5647341

[32] Pizzagalli DA. Depression, stress, and anhedonia: Toward a synthesis and integrated model. Annu Rev Clin Psychol. 2014 Mar;10(1):393–423. doi: 10.1146/annurev-clinpsy-050212-185606 24471371 PMC3972338

[33] Stanton CH, Holmes AJ, Chang SWC, Joormann J. From Stress to Anhedonia: Molecular Processes through Functional Circuits. Trends Neurosci. 2019;42(1):23–42. doi: 10.1016/j.tins.2018.09.008 30327143 PMC6344037

[34] Cooper JA, Arulpragasam AR, Treadway MT. Anhedonia in depression: biological mechanisms and computational models. Curr Opin Behav Sci. 2018 Aug;22:128–35. doi: 10.1016/j.cobeha.2018.01.024 29503842 PMC5828520

[35] Bestbier L, Williams TI. The immediate effects of deep pressure on young people with autism and severe intellectual difficulties: Demonstrating individual differences. Occup Ther Int. 2017;2017(1). 10.1155/2017/7534972 PMC5612681 29097980

[36] Masson HL, Pillet I, Amelynck S, Van De Plas S, Hendriks M, Op De Beeck H, et al. Intact neural representations of affective meaning of touch but lack of embodied resonance in autism: a multi-voxel pattern analysis study. Mol Autism. 2019;10(1):1–14. 31798816 10.1186/s13229-019-0294-0 PMC6881998

[37] Kaiser MD, Yang DYJ, Voos AC, Bennett RH, Gordon I, Pretzsch C, et al. Brain Mechanisms for Processing Affective (and Nonaffective) Touch Are Atypical in Autism. Cereb Cortex. 2016;26(6):2705–14. doi: 10.1093/cercor/bhv125 26048952 PMC4869810

[38] Haggarty CJ, Malinowski P, McGlone FP, Walker SC. Autistic traits modulate cortical responses to affective but not discriminative touch. Eur J Neurosci. 2020;51(8):1844–55. doi: 10.1111/ejn.14637 31793072

[39] Croy I, Geide H, Paulus M, Weidner K, Olausson H. Affective touch awareness in mental health and disease relates to autistic traits–An explorative neurophysiological investigation. Psychiatry Res. 2016;245:491–6. doi: 10.1016/j.psychres.2016.09.011 27639880

[40] Voos AC, Pelphrey KA, Kaiser MD. Autistic traits are associated with diminished neural response to affective touch. Soc Cogn Affect Neurosci. 2013;8(4):378–86. doi: 10.1093/scan/nss009 22267520 PMC3624948

[41] Baron-Cohen S, Wheelwright S, Skinner R, Martin J, Clubley E. The autism-spectrum quotient (AQ): evidence from Asperger syndrome/high-functioning autism, males and females, scientists and mathematicians.[erratum appears in J Autism Dev Disord 2001 Dec;31(6):603]. J Autism Dev Disord. 2001;31(1):5–17. 11439754 10.1023/a:1005653411471

[42] Kroenke K, Spitzer RL, Williams JBW. The PHQ-9: Validity of a brief depression severity measure. J Gen Intern Med. 2001;16(9):606–13. doi: 10.1046/j.1525-1497.2001.016009606.x 11556941 PMC1495268

[43] Cohen S, Solis J, Kamarck T, Mermelstein R. A global measure of perceived stress. J Health Soc Behav. 1983;24:386–96. 6668417

[44] Trotter P, Belovol E, McGlone F, Varlamov A. Validation and psychometric properties of the Russian version of the Touch Experiences and Attitudes Questionnaire (TEAQ-37 Rus). PLoS One. 2019;13(12). 10.1371/journal.pone.0206905 PMC6292699 30543628

[45] Nagy E. Sharing the moment: The duration of embraces in humans. J Ethol. 2011;

[46] Trotter PD, Smith SA, Moore DJ, O’Sullivan N, McFarquhar MM, McGlone FP, et al. Acute tryptophan depletion alters affective touch perception. Psychopharmacology (Berl) [Internet]. 2022;239(9):2771–85. Available from: doi: 10.1007/s00213-022-06151-3 35554625 PMC9385795

[47] Velleman PF, Wilkinson L. Nominal, ordinal, interval, and ratio typologies are misleading. Am Stat. 1993;47(1):65–72.

[48] Mircioiu C, Atkinson J. A comparison of parametric and non-parametric methods applied to a Likert scale. Pharmacy. 2017;5(2):26. doi: 10.3390/pharmacy5020026 28970438 PMC5597151

[49] Bates D, Mächler M, Bolker BM, Walker SC. Fitting linear mixed-effects models using lme4. J Stat Softw. 2015;67(1):1–48.

[50] R Core Team. R: A language and environment for statistical computing. [Internet]. Vienna, Austria.: R Foundation for Statistical Computing; 2013. Available from: http://www.r-project.org/

[51] Fox J, Weisberg S. CAR—An R Companion to Applied Regression. Thousand Oaks CA: Sage. 2019. 2016 p.

[52] Lenth VL. Emmeans. 2021.

[53] Morrison I, Bjornsdotter M, Olausson H. Vicarious responses to social touch in posterior insular cortex are tuned to pleasant caressing speeds. J Neurosci. 2011/07/01. 2011;31(26):9554–62. doi: 10.1523/JNEUROSCI.0397-11.2011 21715620 PMC6623148

[54] Löken LS, Wasling HB, Olausson H, McGlone F, Wessberg J. A topographical and physiological exploration of C-tactile afferents and their response to menthol and histamine. J Neurophysiol. 2022; doi: 10.1152/jn.00310.2021 35020516 PMC9190740

[55] Schirmer A, Chiu MH, Croy I. More Than One Kind: Different Sensory Signatures and Functions Divide Affectionate Touch. Emotion. 2021;Advance on. doi: 10.1037/emo0000966 34435843

[56] Watkins RH, Dione M, Ackerley R, Wasling HB, Wessberg J, Loken LS. Evidence for sparse C-tactile afferent innervation of glabrous human hand skin. J Neurophysiol. 2021 Jan;125(1):232–7. doi: 10.1152/jn.00587.2020 33296618

[57] Lee YS, Sehlstedt I, Olausson H, Jung WM, Wallraven C, Chae Y. Visual and physical affective touch delivered by a rotary tactile stimulation device: A human psychophysical study. Physiol Behav. 2018; doi: 10.1016/j.physbeh.2017.12.022 29274350

[58] Löken LS, Evert M, Wessberg J. Pleasantness of touch in human glabrous and hairy skin: Order effects on affective ratings. Brain Res. 2011;1417:9–15. doi: 10.1016/j.brainres.2011.08.011 21907328

[59] Ackerley R, Carlsson I, Wester H, Olausson H, Backlund Wasling H. Touch perceptions across skin sites: Differences between sensitivity, direction discrimination and pleasantness. Front Behav Neurosci. 2014; doi: 10.3389/fnbeh.2014.00054 24600368 PMC3928539

[60] Sehlstedt I, Ignell H, Wasling HB, Ackerley R, Olausson H, Croy I. Gentle touch perception across the lifespan. Psychol Aging. 2016;31(2):176–84. doi: 10.1037/pag0000074 26950227

[61] Haggarty CJ, Moore DJ, Trotter PD, Hagan R, McGlone FP, Walker SC. Vicarious ratings of social touch the effect of age and autistic traits. Sci Rep. 2021; doi: 10.1038/s41598-021-98802-2 34588542 PMC8481497

[62] Perini I, Gustafsson PA, Igelström K, Jasiunaite-Jokubaviciene B, Kämpe R, Mayo LM, et al. Altered relationship between subjective perception and central representation of touch hedonics in adolescents with autism-spectrum disorder. Transl Psychiatry. 2021; doi: 10.1038/s41398-021-01341-7 33866324 PMC8053196

[63] Russo V, Ottaviani C, Spitoni GF. Affective touch: A meta-analysis on sex differences. Neurosci Biobehav Rev. 2020;108(November 2018):445–52. 31614152 10.1016/j.neubiorev.2019.09.037

[64] Walker SC, Trotter PD, Swaney WT, Marshall A, Mcglone FP. C-tactile afferents: Cutaneous mediators of oxytocin release during affiliative tactile interactions? Neuropeptides. 2017;64:27–38. doi: 10.1016/j.npep.2017.01.001 28162847

[65] Portnova G V., Proskurnina E V., Sokolova S V., Skorokhodov I V., Varlamov AA Perceived pleasantness of gentle touch in healthy individuals is related to salivary oxytocin response and EEG markers of arousal. Exp Brain Res. 2020; doi: 10.1007/s00221-020-05891-y 32719908

[66] Ditzen B, Neumann ID, Bodenmann G, von Dawans B, Turner RA, Ehlert U, et al. Effects of different kinds of couple interaction on cortisol and heart rate responses to stress in women. Psychoneuroendocrinology. 2007; doi: 10.1016/j.psyneuen.2007.03.011 17499441

[67] Feldman R, Singer M, Zagoory O. Touch attenuates infants’ physiological reactivity to stress. Dev Sci [Internet]. 2010;13(2):271–8. Available from: http://doi.wiley.com/10.1111/j.1467-7687.2009.00890.x 20136923 10.1111/j.1467-7687.2009.00890.x

[68] Manzotti A, Cerritelli F, Esteves JE, Lista G, Lombardi E, La Rocca S, et al. Dynamic touch reduces physiological arousal in preterm infants: A role for c-tactile afferents? Dev Cogn Neurosci. 2019;39:100703. 31487608 10.1016/j.dcn.2019.100703 PMC6969366

[69] Pawling R, Cannon PR, McGlone FP, Walker SC. C-tactile afferent stimulating touch carries a positive affective value. PLoS One. 2017;12(3). doi: 10.1371/journal.pone.0173457 28282451 PMC5345811

[70] Walker SC, Marshall A, Pawling R. Psychophysiology and motivated emotion: testing the affective touch hypothesis of C-tactile afferent function. Current Opinion in Behavioral Sciences. 2022.

[71] Pawling R, Trotter PD, McGlone FP, Walker SC. A positive touch: C-tactile afferent targeted skin stimulation carries an appetitive motivational value. Biol Psychol. 2017;129:186–94. doi: 10.1016/j.biopsycho.2017.08.057 28865933

