# Supplementary material for: Hold me or stroke me? Individual differences in static and dynamic affective touch
Source: PLoS One. 2023 May 23;18(5):e0281253. doi: 10.1371/journal.pone.0281253 (PMC10204953; doi:10.1371/journal.pone.0281253)
Supplement: S1 File — This file contains the results of this analysis, as well as the figures depicting the effect of force and velocity on intensity ratings. (PDF) [file pone.0281253.s001.pdf]

Hold me or stroke me? Individual differences in static and dynamic affective touch

S. Hasan Ali, Adarsh D. Makdani, Maria I. Cordero, Aspasia E. Paltoglou, Andrew G. Marshall, Martyn J. McFarquhar, Francis P. McGlone, Susannah C. Walker & Paula D. Trotter

Supplementary materials

Results

Analysis of intensity ratings for directly experienced robotic touch

The interaction between force and velocity was not significant ($F(10,1609.0) = 0.79$, $p = .636$). The main effect of force was significant ($F(2,1609.0) = 663.90$, $p < .001$). Pairwise comparisons identified 1.5 N touch to be rated as significantly more intense than 0.4 N touch ($t(1609) = 19.16$, $p < .001$) and 0.05N touch ($t(1609) = 36.42$, $p < .001$). Additionally, 0.4 N touch was rated as significantly more intense than 0.05 N touch ($t(1609) = 17.28$, $p < .001$). Thus, as would be expected, perceived intensity increased linearly with increasing force.

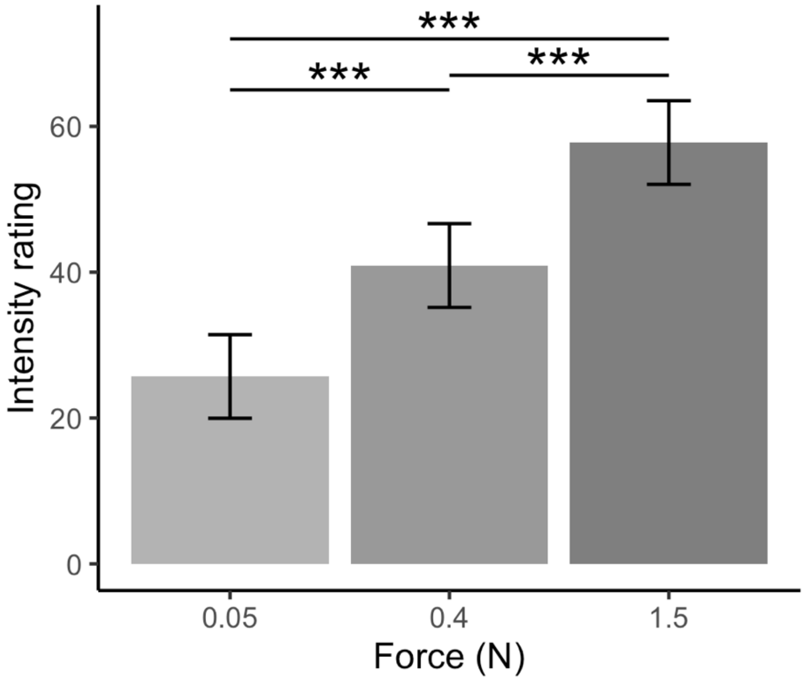

Fig S1. Effect of force on intensity ratings for directly experienced robotic touch to the ventral forearm.

Means ± 95% confidence intervals are shown. Touch applied at a force of 1.5 N was perceived as significantly more intense than 0.4 and 0.05N touch (*p*s < .001). Additionally, touch applied at 0.4 N was rated as significantly more intense than 0.05 N touch (*p* < .001).

The main effect of velocity was significant ($F(5,1609.1) = 42.67$, $p < .001$). Pairwise comparisons identified static touch was rated significantly less intense than all other velocities (*t*s(1609) ≥ 7.60, *p*s < .001). Additionally, touch applied at 0.3 cm/s was rated significantly less intense than all other dynamic touches (*t*s(1609) ≥ 3.39, *p*s ≤ .001). There were no significant differences in intensity ratings for touch applied at 1 cm/s, 3 cm/s, 10 cm/s and 30 cm/s (*t*s(1609) ≤ 1.62, *p*s ≥ .156). Thus, touch delivered at a relatively high force (1.5 N) and a medium to fast stroking velocity (1-30 cm/s) was perceived as most intense.

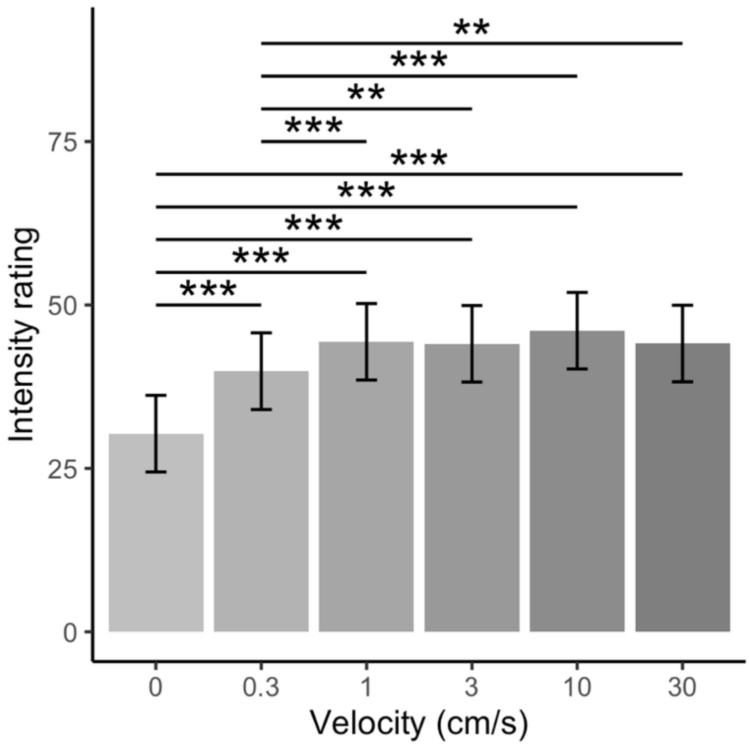

Fig S2. Effect of velocity on intensity ratings for directly experienced robotic touch to the ventral forearm.

Means ± 95% confidence intervals are shown. Static (0 cm/s) touch was rated as significantly less intense than all other velocities (*p*s < .001). Touch applied at 0.3 cm/s was rated significantly less intense than all other dynamic touches (*p*s ≤ .001). There were no significant differences in intensity ratings for touch applied at 1 cm/s, 3 cm/s, 10 cm/s and 30 cm/s (*p*s ≥ .156).
